# Bioimaging of sense organs and the central nervous system in extant fishes and reptiles in situ: A review

**DOI:** 10.1002/ar.25566

**Published:** 2024-09-02

**Authors:** Shaun P. Collin, Kara E. Yopak, Jenna M. Crowe‐Riddell, Victoria Camilieri‐Asch, Caroline C. Kerr, Hope Robins, Myoung Hoon Ha, Annalise Ceddia, Travis L. Dutka, Lucille Chapuis

**Affiliations:** ^1^ School of Agriculture, Biomedicine and Environment La Trobe University Bundoora Victoria Australia; ^2^ Department of Biology and Marine Biology University of North Carolina Wilmington Wilmington North Carolina USA; ^3^ School of Biological Sciences The University of Adelaide Adelaide South Australia Australia; ^4^ Max Planck Queensland Centre for the Materials Science of Extracellular Matrices Queensland University of Technology Kelvin Grove Queensland Australia; ^5^ School of Biological Sciences University of Bristol Bristol UK; ^6^ Leigh Marine Laboratory, Institute of Marine Science University of Auckland Leigh New Zealand

**Keywords:** brain, CNS, ear, eye, MRI, olfactory rosette, vomeronasal organ, μCT

## Abstract

Bioimaging is changing the field of sensory biology, especially for taxa that are lesser‐known, rare, and logistically difficult to source. When integrated with traditional neurobiological approaches, developing an archival, digital repository of morphological images can offer the opportunity to improve our understanding of whole neural systems without the issues of surgical intervention and negate the risk of damage and artefactual interpretation. This review focuses on current approaches to bioimaging the peripheral (sense organs) and central (brain) nervous systems in extant fishes (cartilaginous and bony) and non‐avian reptiles *in situ*. Magnetic resonance imaging (MRI), micro‐computed tomography (μCT), both super‐resolution track density imaging and diffusion tensor‐based imaging, and a range of other new technological advances are presented, together with novel approaches in optimizing both contrast and resolution, for developing detailed neuroanatomical atlases and enhancing comparative analyses of museum specimens. For MRI, tissue preparation, including choice of fixative, impacts tissue MR responses, where both resolving power and signal‐to‐noise ratio improve as field strength increases. Time in fixative, concentration of contrast agent, and duration of immersion in the contrast agent can also significantly affect relaxation times, and thus image quality. For μCT, the use of contrast‐enhancing stains (iodine‐, non‐iodine‐, or nanoparticle‐based) is critical, where the type of fixative used, and the concentration of stain and duration of staining time often require species‐specific optimization. Advanced reconstruction algorithms to reduce noise and artifacts and post‐processing techniques, such as deconvolution and filtering, are now being used to improve image quality and resolution.

## Graphical abstract

Bioimaging of the sense organs and brain of fishes and reptiles. Left panel: 3D reconstruction of the head and brain of the deep‐sea viperfish *Chauliodus sloani* following diceCT. Right panel: A 3D reconstruction of a 70‐day‐old embryo head of the bearded dragon *Pogona vitticeps* following diceCT, showing the position of the segmented brain within the head.

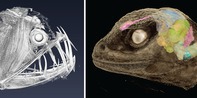

## 1 INTRODUCTION

*In situ* bioimaging of the peripheral and central nervous systems (CNSs) is increasingly being used to assess the levels of morphological variation and adaptation to predict structure–function relationships in light of phylogeny, ontogeny, and plasticity. Fishes and reptiles are two diverse (and speciose) groups of vertebrates, which are subject to an array of sensory demands based on each species' environmental microhabitat and represent important taxa for comparative neuroanatomical studies. A range of bioimaging techniques, including micro‐computed tomography (μCT), magnetic resonance imaging (MRI), as well as super‐resolution track density imaging (TDI) and diffusion tensor‐based imaging (DTI) are reviewed, including how they are being used to render high‐resolution 2D and 3D models of the peripheral and CNSs. Current approaches and technologies for *in situ* bioimaging are presented to optimize investigations of neuroanatomy, connectivity, and brain shape in the nervous system of a range of extant species of fishes and reptiles. These approaches may also assist in the quantification of the relative size of peripheral sense organs and central brain regions, targeting specific regions for tract tracing studies, localizing neural targets for electrophysiological recording and hodological mapping of brain regions. Bioimaging studies are improving our understanding of the evolutionary drivers of sensory adaptation and brain plasticity in key taxa and creating exciting opportunities to incorporate large collections of museum specimens and allow these to be electronically accessible.

## 2 ADVANTAGES OF IN SITU BIOIMAGING IN COMPARATIVE NEUROBIOLOGY

Advances in our understanding of neuroanatomy and brain circuitry in fishes and reptiles are enabled by major technological advancements in the last century that have allowed us to visualize and characterize nervous system tissue at a variety of levels of biological organization. These range from the assessment of gross morphology to the description of intricate three‐dimensional (3D) neuroanatomical structures and regional connectivity and even the mapping of molecular features in neurons and glial cells (Smeets et al., 1983, 1986; ten Donkelaar, 1998; Wullimann et al., 1996). However, many current neuroanatomical methods used for the characterization of the brain and other soft tissues are invasive, often requiring degradation or destruction of the specimen or sample. While we gain cellular and/or molecular detail, we can lose precision with regard to the relative position of anatomical structures, as well as contend with shrinkage artifacts due to sectioning and staining (Ullmann, Cowin, & Collin, 2010a), shape distortions, and labor‐intensive tissue processing (Nieman, 2005). In addition to the potential loss of important anatomical information, these challenges render the assessment of rare, endangered, or invaluable museum specimens impractical, which may limit the taxonomic breadth of comparative studies.

In contrast to surgical methods exposing the peripheral and CNSs, bioimaging enables the acquisition of high‐resolution images *in vivo* or *ex vivo*. Bioimaging also provides information on the size, shape, and spatial relationships of neuronal brain structures when acquired as an isotropic, three‐dimensional dataset along any stereotaxic plane. However, when peripheral sense organs, that is, eyes, nose, and ears, and their afferent cranial nerves, are also imaged, it is possible to quantitatively assess the relative importance of different sensory modalities and make predictions of the evolutionary drivers of neural adaptations to environmental factors.

Fishes (>32,000 extant species) and non‐avian reptiles (>10,000 extant species) are extraordinarily diverse groups encompassing over half of all vertebrate diversity. Fishes and reptiles occupy a multitude of the earth's ecosystems and possess a diversity of sensory modalities, some of which are not found in many other vertebrate taxa. In addition to visual (eyes), non‐visual (photoreceptive and non‐image‐forming tissue), chemoreceptive (olfactory and gustatory organs), and auditory (inner ears) modalities, some species of fishes and reptiles possess a lateral line (neuromasts in both cartilaginous and bony fishes), electroreception (ampullae of Lorenzini in cartilaginous fishes and tuberous electroreceptors in a restricted group of bony fishes), and thermoreception (e.g., thermosensitive pit organs in snakes). Both groups have indeterminate growth or continue to grow throughout life (Dutta, 1994; Matta et al., 2017; Frýdlov et al., 2020, although see Frýdlov et al., 2019), where neurogenesis persists into adulthood (Otteson et al., 2002). Therefore, these two groups represent important model vertebrate taxa for comparative neuroanatomical studies of plasticity at evolutionary, developmental, and environmental scales. Although there have been recent advances in bioimaging of the brain and nervous system of amphibians (see Chai & Sailler, 2023; Fidalgo et al., 2018; Lanctôt et al., 2021; Ruffins & Jacobs, 2011; Tesarová, 2022; Tyszka et al., 2005) due to the focus of our collaborative expertise on fishes and reptiles, we have focussed only on these two speciose groups.

The following sections describe the techniques of MRI and micro‐computed tomography (μCT) including the methods for optimizing both resolution and contrast, the most recent technological advances, and the uses of these innovative technologies in comparative neuroanatomy. Although of high importance, the imaging of extinct (fossilized) fishes and reptiles is not included. However, see Clement et al. (2015, 2016) in fishes and Roese‐Miron et al. (2024), Sobral (2023) and Bazzana et al. (2023) in reptiles for recent approaches to generating cranial endocasts to trace the evolution of sense organs and the brain in extinct representatives of these two taxa.

## 3 MAGNETIC RESONANCE IMAGING

**FIGURE 1:**
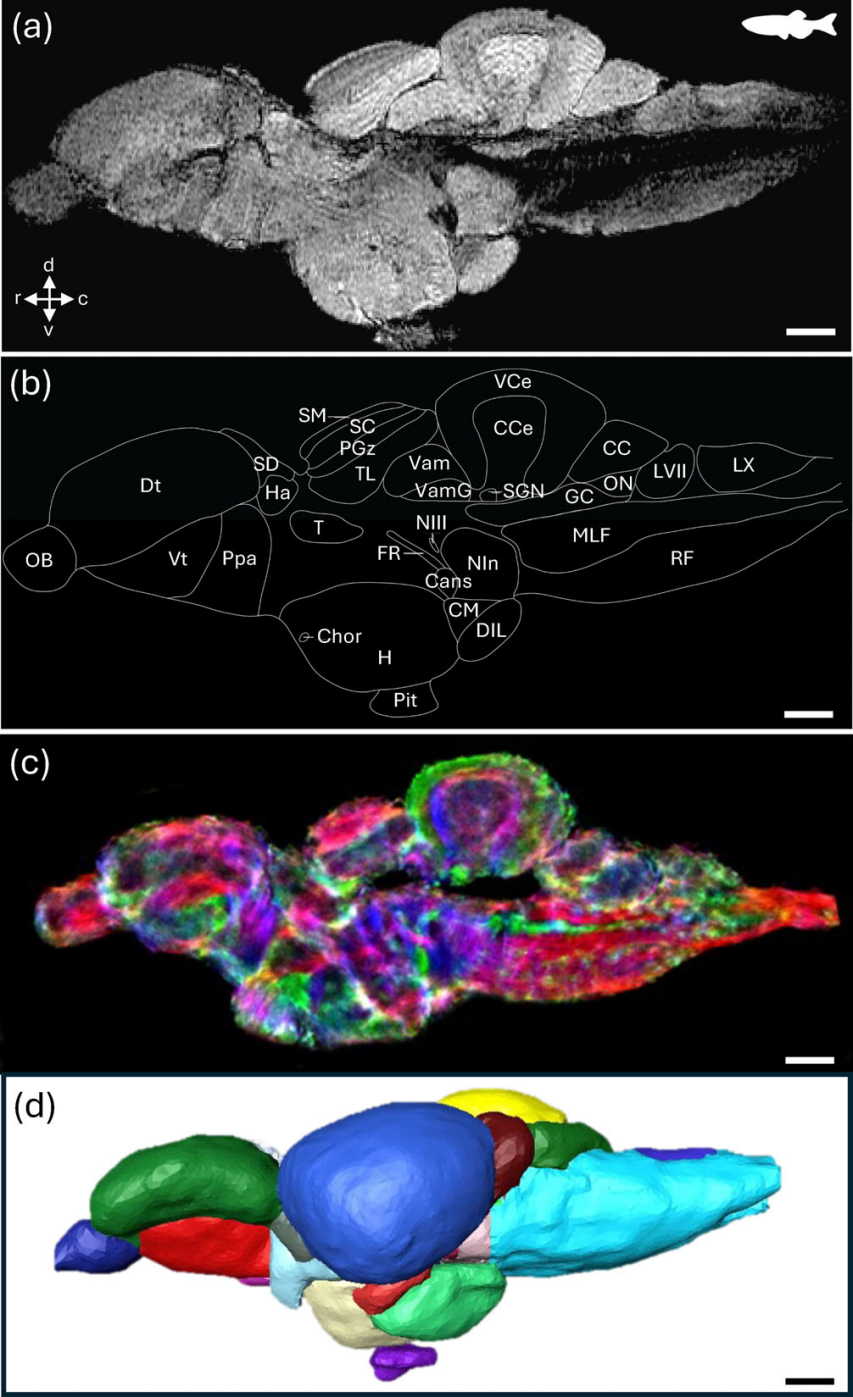
Magnetic resonance images and segmentation of the brain of the freshwater zebrafish *Danio rerio*. (a) A T2*‐weighted scan of the brain of *D. rerio* in sagittal section along the midline. (b) Schematic drawing of the major brain structures seen in (a). Identified structures: Cans, commissura ansulata; CC, cerebellar crest; CCe, corpus cerebelli; Chor, commisura horizontalis; CM, corpus mammilare; DIL, nucleus diffusus lobi inferioris hypothalami; Dt, area dorsalis telencephali; FR, fasciculus retroflexus; GC, griseum central; H, hypothalamus; Ha, habenula; LVI, lobus facialis; LX, lobus vagi; MLF, fasciculus longitudinalis medialis; NIn, nucleus interpeduncularis; NIII, nervus oculomotorius; OB, bulbus olfactorius; ON, nucleus octavolateralis; PGz, stratum periventriculare tecti optici; Pit, glandula pituitaria; Ppa, nucleus praeopticus parvocellularis pars anterior; RF, formatio reticularis; SC, stratum centrale; SD, saccus dorsalis; SGN, nucleus gustatorius secundarius; SM, stratum marginale; T, thalamus; TL, torus longitudinalis; Vam, valvula cerebelli, pars medialis; VamG, valvula cerebelli, pars medialis, granular layer; VCe, vestibulolateralis lobe of CCe; Vt, area ventralis telencephali. From Ullmann, Cowin, et al. (2010c), Ullmann et al. (2015). (c) Sagittal section showing directionally‐encoded color short track diffusion tensor image maps (5 μm isotropic resolution) generated from diffusion‐weighted imaging data acquired on a 16.4‐T scanner (at 48 μm isotropic resolution). Color‐coding indicates the local fiber orientation (with red rostral–caudal, green medial–lateral, and blue dorsal–ventral. (d) Three‐dimensional representation of the segmented brain regions in lateral view. See Ullmann, Cowin, et al. (2010c) (Video 1) for an animated video of the segmented brain atlas. Scale bar = 0.5 mm. c, caudal; d, dorsal; r, rostral; v, ventral. (a), (b), and (d) reproduced with permission from Ullmann, Cowin, et al. (2010c). (c) Reproduced with permission from Ullmann et al. (2015).

**FIGURE 2:**
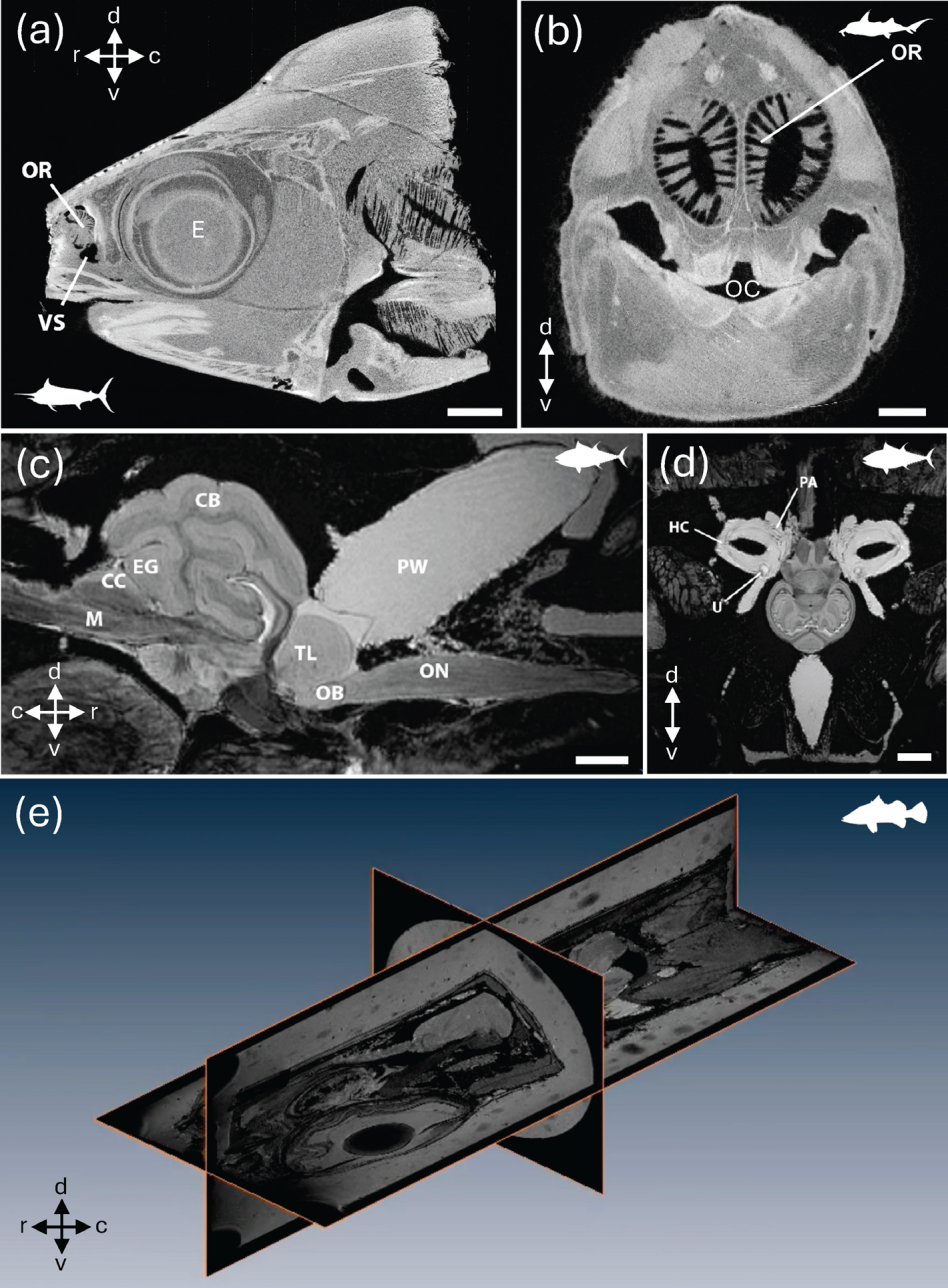
2D and 3D images of a range of fish species. (a) Sagittal X‐ray image of the head of the black marlin *Istiompax indica* showing its large eye (E), pleated olfactory rosette (OR), and ventilatory olfactory sac (VS). (b) Axial X‐ray image of the head of the Australian ghost shark *Callorhinchus milii*, showing the olfactory rosette (OR) and its associated lamellae sitting dorsal of the oral cavity (OC). Acquired at the Imaging and Medical Beamline of the Australian Synchrotron. (c, d). Sagittal (c) and axial (d) MRI sections through the cranium of the Southern bluefin tuna *Thunnus maccoyii*, acquired with a whole body Siemens Magnetom 7 T at The University of Melbourne Brain Centre Imaging Unit in Australia. CB, cerebellum; CC, cerebellar crest; EG, eminentia granularis; M, medulla; TL, telencephalon; OB, olfactory bulb; ON, olfactory nerve; PW, pineal window; U, utricle; HC, horizontal canal; PA, posterior ampulla. (e). Three‐dimensional representation of the segmented brain regions of the barramundi *Lates calcarifer* in lateral view following transcardial perfusion with Magnevist1 (Gd‐DTPA, Berlex, Wayne, NJ) and 4% paraformaldehyde in 0.1 M phosphate buffer. See Video 2 for the animation. All magnetic resonance images were acquired on a Bruker (Ettlingen, Germany) AV II spectrometer running ParaVision 4, interfaced to a 16.4 T vertical magnet with a micro 2.5 gradient system and a 25 mm (ID) birdcage radiofrequency coil. See Ullmann, Cowin, & Collin, 2010b) for more information. Scale bar = 1 cm (a); 1 cm (b); 0.5 cm (c); 1 cm (d). c, caudal; d, dorsal; r, rostral; v, ventral. Animation in (e) was constructed using data from Ullmann, Cowin, and Collin (2010b).

**FIGURE 3:**
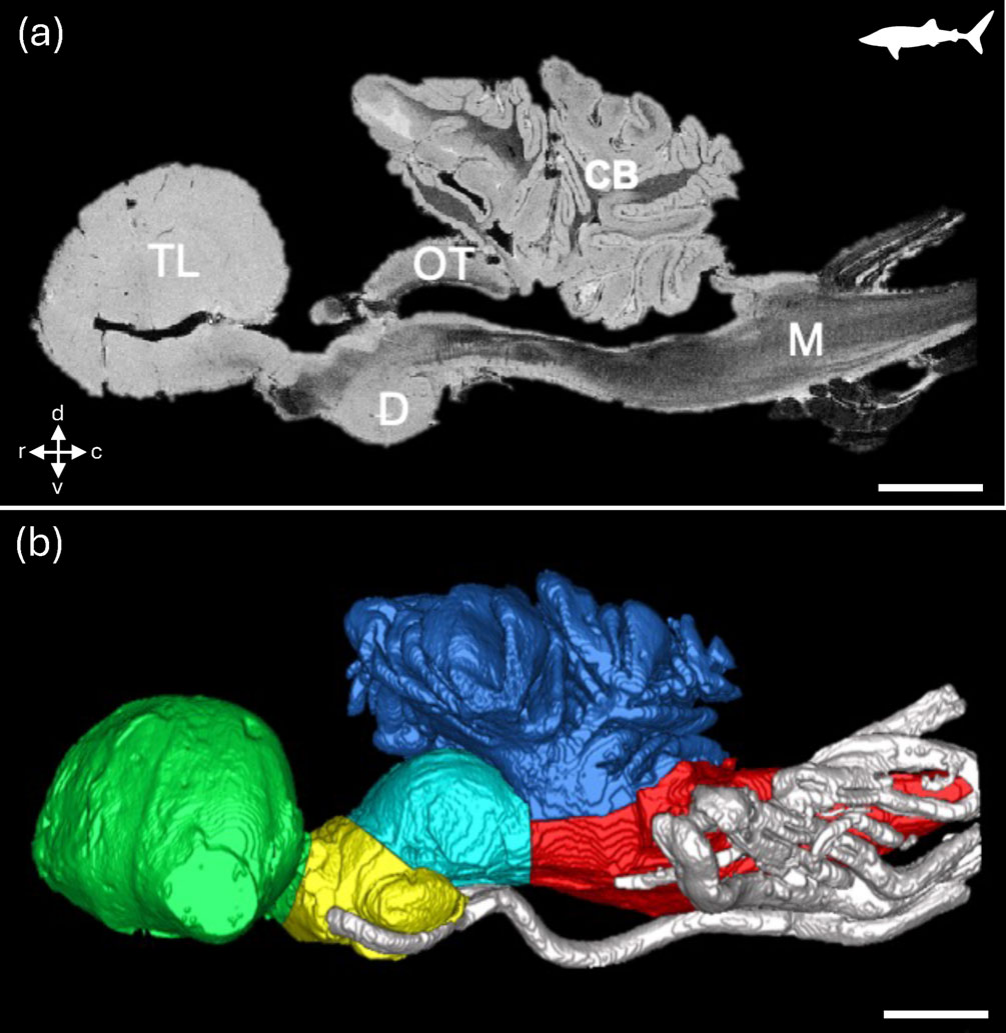
T1‐weighted 3D fast spoiled gradient echo (FSPGR) MRI image of the brain of the whale shark *Rhincodon typus* showing a sagittal slice of an excised brain from a juvenile specimen (a) and a digital segmentation of the major structures of the brain and cranial nerves (b). CB, cerebellum; D, diencephalon; M, medulla oblongata; OT, optic tectum. c, caudal; d, dorsal; r, rostral; v, ventral. Scale bar = 1 cm. (a) and (b) Reproduced with permission from Yopak and Frank (2009).

**FIGURE 4:**
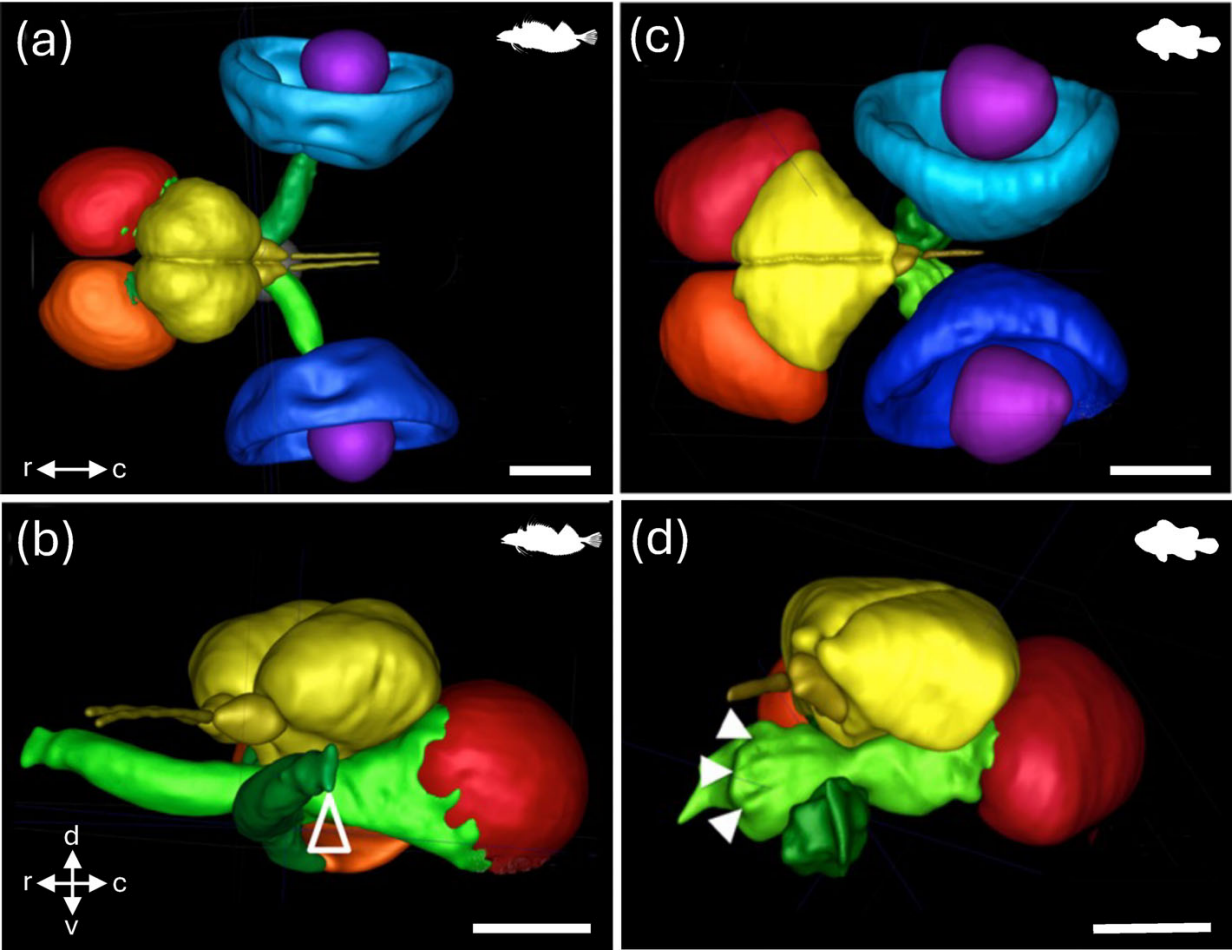
MRI segmentations of the brain and eyes in the teleosts, the triplefin blenny *Tripterygion melanurus*, and the clownfish *Amphiprion ocellaris*. (a, b). Dorsal (a) and lateral (b) views of *A. ocellaris*. (c, d). Dorsal (c) and lateral (d) views of *T. melanurus*. Following fixation in 4% paraformaldehyde in 0.1 M phosphate buffer and immersion in 0.25% of 1 M Gadovist® (Bayer AG, 51373 Leverkusen, Germany, scans were acquired overnight on a 16.4 T Ultrashield™ Plus 700 WB Avance NMR spectrometer running ParaVision® 5.1 software. Retinae (blue), lenses (purple), optic nerves and tracts (green), optic tecta (red), telencephalon, olfactory nerves and bulbs, and diencephalon (yellow). The open arrowhead in (b) depicts the optic disc (retina not shown). Closed arrowheads in (d) depict the folds of the pleated optic nerve. c, caudal; d, dorsal; r, rostral; v, ventral. Scale bars = 1 mm. All panels are reproduced with permission from Fritsch et al. (2017).

Magnetic resonance imaging (MRI) is an emerging method in non‐human comparative neuroscience and allows for non‐invasive acquisition of high‐resolution (from 10 μm), 3D data of soft tissues *in situ*. These methods have even been applied *in vivo* in studies of fish physiology, allowing for the quantification of bulk flow and diffusion (Bock et al., 2002; Mark et al., 2002). While MRI has been extensively optimized for human and mammalian veterinary medicine (Elliott & Skerritt, 2010; Kiessling et al., 2011), there has been a shift toward the development of these methods for non‐invasive visualization and quantification of neuroanatomical features in fishes and reptiles, ranging from whole‐body imaging to higher‐resolution scans of the peripheral and CNSs (Figures 1, 2, 3, 4).

Magnetic resonance imaging utilizes a strong magnetic field, which can vary in its magnetic field strength and is measured in tesla (T) or gauss (G), and radiofrequency waves to create high‐resolution, cross‐sectional images of soft tissue structures. In brief, the magnetic field aligns hydrogen atoms (i.e., protons) in the imaged object, capitalizing on the fact that most organismal tissue is comprised of a high proportion of water. The protons absorb the radiofrequency waves, generating a detectable signal that is transmitted to a radiofrequency coil and processed as a series of sliced images by a computer. Therefore, the spatial distribution of varying water content can be mapped between tissue types. In addition to proton density, recovery (longitudinal relaxation time, T_1_) and decay of the MRI signal (transverse relaxation time, T_2_) (Callaghan, 1991) also vary between soft tissue structures. Taken together, the generation of images with high contrast between tissues with different relaxation times is the most common method of anatomical imaging. This includes T_1_‐weighted imaging (which emphasizes variation in T_1_), which is often used to maximize contrast between gray and white matter in the brain by suppressing the water signal (Yopak et al., 2016; Yopak & Frank, 2009), and distinguishing among cranial structures such as eye, cartilage, and stomach (Waller et al., 1994; Figure 1). On the other hand, T_2_‐weighted imaging (which emphasizes variation in T_2_) often optimizes variation between tissue and water. Both T_1_‐weighted (González et al., 2023; Libourel et al., 2018; Peele et al., 2023; Yopak et al., 2019; Yopak & Frank, 2009) and T_2_‐weighted (Billings et al., 2020; Foss et al., 2022; Hoops et al., 2018; Jiménez et al., 2024; Kabli et al., 2006), imaging has been successfully applied to nervous system tissue in studies of comparative brain morphology in fishes and reptiles. Although it has more common applications in diagnostic research, several studies have also employed T_2_*‐weighted imaging in fishes (Ullmann, Cowin, & Collin, 2010b; Ullmann, Cowin, et al., 2010c, Figure 1) and reptiles (Hoops et al., 2021; Hoops, Vidal‐Garcia, et al., 2017, Figure 4) in the development of MR‐based brain atlases, which includes T_2_ effects in combination with magnetic field inhomogeneities that arise from susceptibility differences between tissues.

**VIDEO 1:** Three‐dimensional representation of the segmented brain regions of *D. rerio* in lateral view. See Ullmann, Cowin, et al. (2010c) for an animated video of the segmented brain atlas.

**VIDEO 2:** Three‐dimensional representation of the segmented brain regions of the barramundi *Lates calcarifer* in lateral view following transcardial perfusion with Magnevist1 (Gd‐DTPA, Berlex, Wayne, NJ) and 4% paraformaldehyde in 0.1 M phosphate buffer.

### 3.1 Optimizing resolution

Image quality can vary greatly between scans and is dependent upon tissue quality (freshness) and sample preparation (reviewed in Berquist et al., 2012; Yopak et al., 2018; Ziegler & Mueller, 2011), as well as magnetic field strength and optimization of scan parameters (see Brown et al., 2014; Kiessling et al., 2011). Tissue preparation, including choice of fixative (Chanet et al., 2009; Ullmann, Cowin, & Collin, 2010b; Waller et al., 1994; Yong‐Hing et al., 2005), freezing and thawing prior to imaging (Nott et al., 1999a, 1999b; Perry et al., 2007), can all have a great impact on the resultant tissue MR response (see Berquist et al., 2012 for best practices for MRI in fishes).

**VIDEO 3:** Avizo animation of the olfactory rosette revealing the two segmented clusters of glomeruli in the right olfactory bulb of the brownbanded bamboo shark *Chiloscyllium punctatum*, suggesting a potentially somatotopic or zonal organization in the integration of primary inputs in the olfactory system of this species.

**VIDEO 4:** Avizo animation of head revealing the central nervous system (brain) of the brownbanded bamboo shark *Chiloscyllium punctatum*, showing the levels of contrast between and within soft tissues.

**VIDEO 5:** Axial X‐ray μCT coronal image stack through the head of *H. ocellatum* at the level of the midbrain.

**FIGURE 5:**
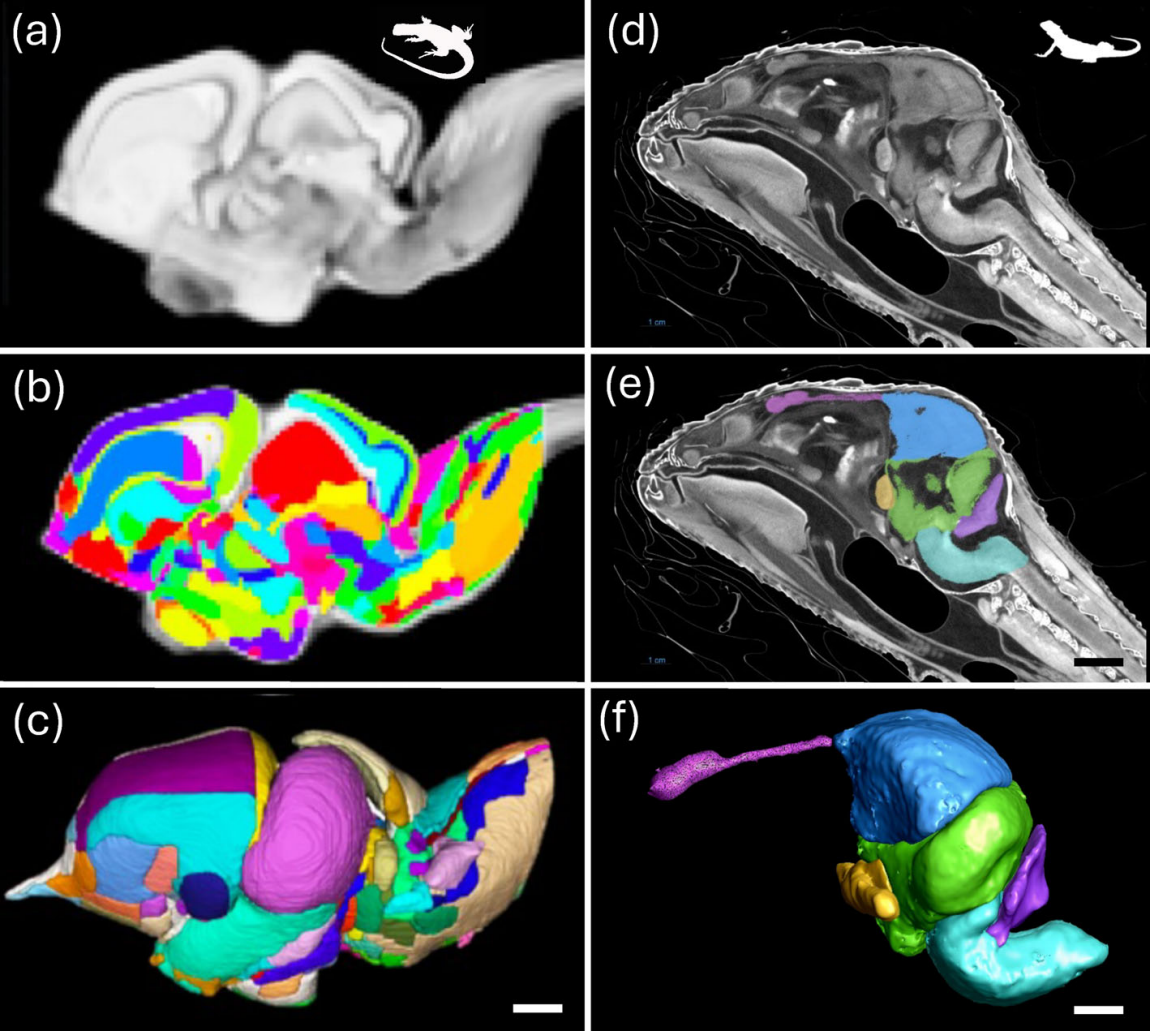
Bioimaging in reptiles using MRI (left) and μCT (right). (a) A T2*‐weighted scan of the tawny dragon *Ctenophorus decresii*, showing a sagittal slice of the brain (a), sagittal section showing the segmentation in 2D (b), and segmentation atlas, in lateral surface view in a 3D render of the segmented regions (c). (d) diceCT scans of the bearded dragon *Pogona vitticeps* showing a sagittal slice of the embryonic brain *in vivo*. (e). Segmentation of the embryonic brain and (f) a 3D render of the segmented regions, both performed using Dragonfly software. Scale bars = 1 mm (a–e) and 2 mm (f). (a–c) Reproduced with permission from permission Hoops et al. (2021).

Spatial resolution, or the ability to resolve two points as distinct, will greatly impact the capacity to resolve the neuroanatomical structure(s) of interest. In MRI (and similarly for μCT, see below), spatial resolution is defined by the size of the individual volume resolution elements, termed a voxel. The size of a voxel is set from the field of view (FOV), slice thickness, and matrix size, with smaller voxel sizes providing higher spatial resolution. An MR image is a combination of pure signal and noise, so the signal‐to‐noise ratio (SNR) is used to describe and evaluate the contrast in an image. Higher SNR can be acquired by either increasing FOV and slice thickness, which increases sampling volume but decreases spatial resolution, or by increasing the strength of the magnetic field. In most clinical and non‐clinical research facilities, the field strength of accessible scanners often ranges from 1.5 to 16.4 T, with variable limitations in the size of specimens that can be imaged. Scanners with lower magnetic field strengths (1.5–4.7 T) have larger usable bores, which allows for whole‐body imaging or scans of aspects of larger‐bodied specimens, such as larger sharks (Figure 3), bony fishes (Figures 1 and 2), and reptiles (Figure 5) (González et al., 2023; Jiménez et al., 2024; Jirak & Janacek, 2017; Lauridsen et al., 2011; Perry et al., 2007; Scadeng et al., 2020; Yopak & Frank, 2009; Ziegler et al., 2014), but often at a cost of spatial resolution (as fine as 90–100 μm, but more often 300–500 μm). However, as field strength increases, the resolving power and SNR improve, allowing for the acquisition of higher‐resolution images with improved contrast. Therefore, small‐animal scanners (with a small bore size), including 7 T or 9.4 T, can produce images with enough resolution to differentiate individual brain regions from whole‐body scans in teleosts (Berquist et al., 2012; Kabli et al., 2006). High‐resolution scans (70–150 μm), together with optimized contrast between gray and white matter, can also enable the identification of major brain nuclei, from whole‐head imaging of smaller sharks and crocodilians (Billings et al., 2020; Peele et al., 2023), the heads of small pelagic teleosts such as the Southern bluefin tuna *Thunnus maccoyii* (Figure 2) or in excised brains from larger animals (Yopak & Frank, 2009; Yopak et al., 2016, 2019, Figure 3). Current protocols in MR microscopy (Tyszka et al., 2005) have produced brain scans at resolutions down to 10–30 μm in small‐bodied specimens like the zebrafish *Danio rerio* (Ullmann, Cowin, et al., 2010c, 2010d, Figure 1), barramundi *Lates calcarifer* (Ullmann, Cowin, & Collin, 2010b, Figure 2), the clownfish *Amphiprion ocellaris* (Figure 4), cleaner wrasse *Labroides dimidiatus*, and the tawny dragon *Ctenophorus decresii* (Hoops et al., 2021; Hoops, Ullmann, et al., 2017; Hoops, Vidal‐Garcia, et al., 2017, Figure 4) using 11.4 T or 16.4 T small‐bore scanners. Spatial resolution is limited by the field strength of MRI scanners (in addition to other factors, including length of the scan, sampling rate, field of view, and SNR, among others), but bore (and coil) size limits the size of the sample that can be scanned, which is an important consideration depending on the specimen in question. For example, Ullmann, Cowin, et al. (2010c) and Hoops et al. (2021) were able to delineate 53 and 224 brain structures in *D. rerio* and C. *decresii*, respectively (Figures 1 and 5).

**VIDEO 6:** Animated segmentation of the brain and both labyrinths in *H. ocellatum*.

**VIDEO 7:** Animation of dorso‐lateral “fly‐through” of a contrast‐enhanced μCT scan of the blunthead tree snake *Imantodes cenchoa* that was stained using 1.25% Lugol's iodine solution and segmented and animated using VG Studio MAX software (Volume Graphics, Heidelberg, Germany) at the University of Michigan Museum of Zoology (Museum No. UMMZ246810).

### 3.2 Optimizing contrast

The ability of the scanner to detect the MR signal in different types of tissues is also highly dependent on optimizing anatomical contrast in the tissue(s) of interest. Gadolinium‐based contrast agents (Weinmann et al., 1984) can enhance the MR responses of tissues by improving the efficiency of T_1_ relaxation of the sample (and to a lesser extent, T_2_), producing a brighter signal in T_1_‐weighted images, and increasing SNR in a shorter scan time (Ullmann, Cowin, et al., 2010d). This can greatly improve image acquisition efficiency, as well as enhance the ability to resolve boundaries between structures, enabling the identification of key brain nuclei. However, some tissues are prone to susceptibility effects, especially at high magnetic field strengths, resulting in local image distortions.

There is a wide range of gadolinium‐based contrast agents employed in studies of comparative neuroanatomy; although not exhaustive, these include ProHance® (Bracco Diagnostics) (Berquist et al., 2012; Peele et al., 2023; Yopak & Frank, 2009); Dotarem® (Guerbet) (Billings et al., 2020; Yopak et al., 2019), Magnevist (Berlex) (Hoops et al., 2018; Hoops et al., 2021; Hoops, Vidal‐Garcia, et al., 2017; Triki et al., 2021; Ullmann, Cowin, & Collin, 2010a), and Optimark® (Mallinckrodt Inc.) (Ullmann, Cowin, et al., 2010d). Ullmann, Cowin, et al. (2010d) performed a comprehensive study on image optimization in the zebrafish brain using seven different fixatives (including 70% ethanol, 4% paraformaldehyde, and 4% glutaraldehyde) and two contrast agents (Magnevist and Optimark), as well as variable durations for fixation and contrast agent concentrations. Time in fixative, concentration of contrast agent, and duration of immersion in the contrast agent can all significantly affect relaxation times, and thus image quality (Ullmann, Cowin, et al., 2010d), with likely variation and the need for optimization between species. There is further work needed to understand the impacts of storage solutions (such as those used in museums) on imaging in fishes and reptiles. While other contrast agents have been used in MR studies, including chromium‐ and manganese‐based (see Ullmann et al., 2015 for review), gadolinium‐based agents remain the most common in comparative neuroscience.

### 3.3 Diffusion MRI

The gold standard for assessing structural connectivity within the CNS is provided by tract‐tracing studies, which investigate the relative number and trajectory of axonal connections within the brain using neuroanatomical tract tracer agents, for example, viral, bacterial, or biotinylated dextran agents (Fletcher et al., 2014; Fritzsch & Collin, 1990; Köbbert et al., 2000). However, TDI and DTI are relatively new, non‐invasive techniques that measure molecular diffusivity of water inside brain tissue using a specialized type of MRI, offering high resolution and contrast (Calamante et al., 2010). Diffusion‐weighted imaging (DWI) measures the thermal Brownian motion of water molecules in brain tissue. Water molecules tend to diffuse more freely along the direction of axonal fascicles with the direction of maximum diffusivity captured in the final image. The mechanisms underlying anisotropic water diffusion appear to be common to all nervous systems (Beaulieu, 2002). TDI integrates data from fiber tracks across large spatial scales to yield image maps encoding information on fiber directionality, displayed in directionally‐encoded colors. Specific fiber bundles can thus be followed using tractography algorithms, providing an index of the connections between particular brain regions and the routes these nerve bundles take. There are only a limited number of studies that have investigated approaches to optimize contrast and resolution using diffusion MRI methods in non‐mammalian vertebrates. In contrast to T1/T2* MRI sequences, TDI is based on measurements in a whole‐brain tractogram generated from DWI data and relies on the inherent structure and connectivity of the neural tissue to provide contrast. The super‐resolution properties of the TDI technique (Calamante et al., 2011) greatly improve the spatial resolution achievable by this MRI method, where the morphological structures visualized (up to 5 μm resolution) on TDI maps are very similar to those images generated using conventional histology. The super‐resolution and the local directional information offered by directionally‐encoded color TDI enables a larger number of brain regions, commissures, and small white matter tracks to be differentiated when compared to conventional MRI and DTI. It has successfully been applied to provide an enhanced characterization of the zebrafish brain (Ullmann et al., 2015, Figure 1c), and offers great promise for comparative studies, providing the opportunity to investigate the connectivity and functional anatomy of the CNS.

## 4 MICRO‐COMPUTED TOMOGRAPHY

Micro‐X‐ray computed tomography (μCT) is a valuable technique for examining the histology of biological tissues *in situ*. In contrast to MRI, X‐ray computed tomography uses the projection of X‐rays around the body of a specimen or 2D radiographs that are processed using reconstruction algorithms to produce 2D cross‐sectional images that are ultimately used to render a 3D image. Micro‐CT is typically less expensive, faster, and tends to offer higher resolution (threshold of 1 μm compared to MRI with a threshold of 10 μm), although this is highly dependent on the size of the region of interest and therefore the field of view.

Due to the large difference in X‐ray attenuation of mineralized tissues, μCT has been widely used to image hard tissues like bones and teeth. Such μCT scanning has generated a plethora of studies that have been used to infer the shape of neuroanatomical structures that are encased in bone in both extant and fossil taxa, that is, brains and ears especially in lizards and snakes (Allemand et al., 2023; Witmer et al., 2008). To extend μCT to visualize the nervous system, the specimen can be stained prior to μCT scanning using contrast‐enhancing staining agents that add density to soft tissues. These agents can be iodine‐based (KI, potassium iodide and I_2_KI, iodine–potassium iodide also known as Lugol's solution), non‐iodine‐based (such as PTA, phosphotungstic acid; PMA, phosphomolybdic acid; and OsO_4_, osmium tetroxide), or nanoparticle‐based (i.e., using Au, Bi, Ta, and Gd nanoparticles) (Koç et al., 2019).

**FIGURE 6:**
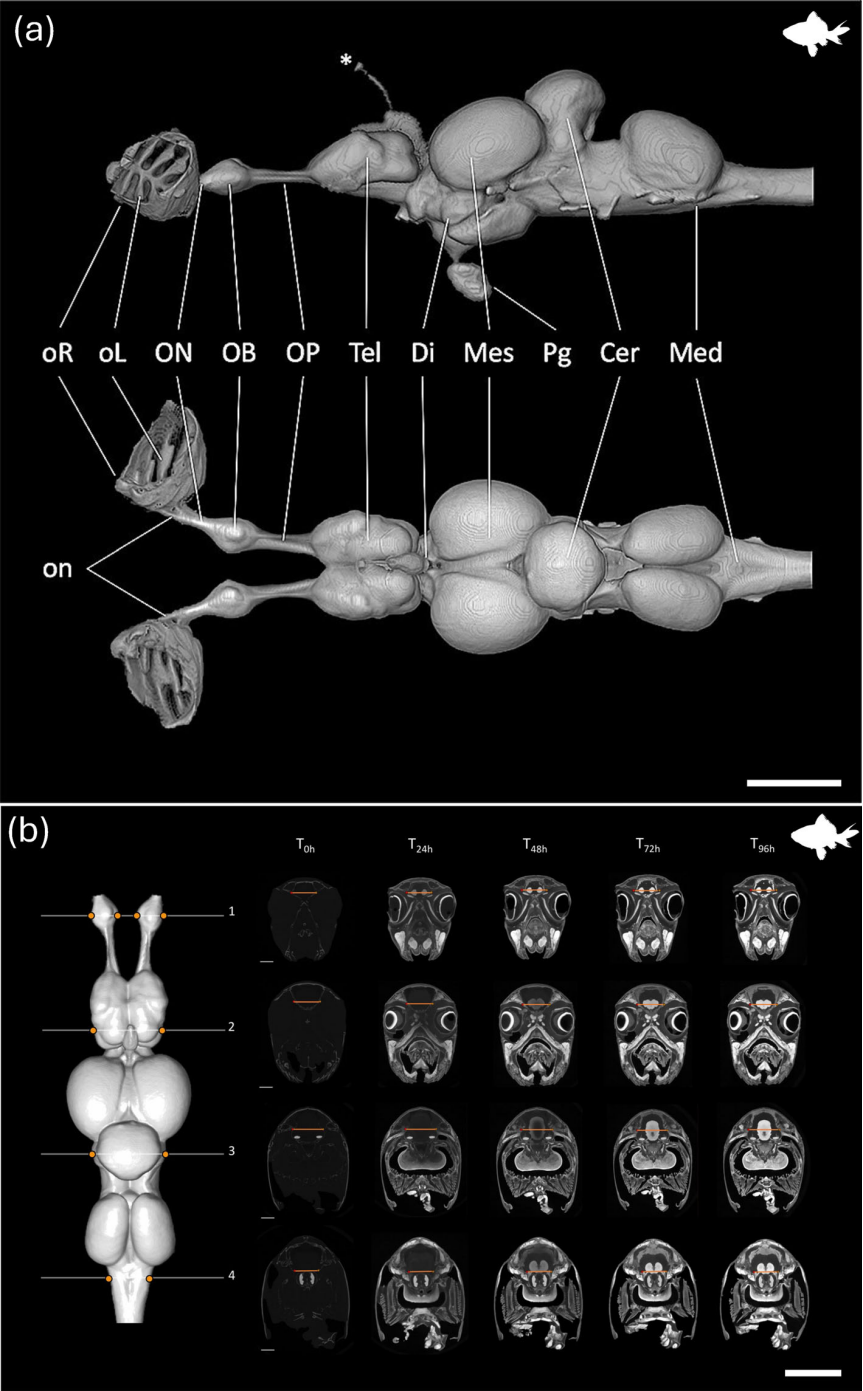
Bioimaging of the nervous system in the goldfish. (a). Lateral view (top) and dorsal view (bottom) of the brain and olfactory peripheral nervous system of the goldfish *Carassius auratus*, segmented from μCT images in Avizo software. *, pineal gland and nerve; oR, olfactory rosette; oL, olfactory lamella; on, primary olfactory nerve bundle; ON, olfactory nerve (cranial nerve I); OB, olfactory bulb; OP, olfactory peduncle; Tel, telencephalon; Di, diencephalon; Mes, mesencephalon; Pg, pituitary gland; Cer, cerebellum; Med, medulla oblongata. c, caudal; d, dorsal; r, rostral; v, ventral. Scale bar = 2 mm. (b) Segmented brain of *C. auratus* in dorsal view (left) and the positions of four sample slices (numbered 1–4 in white) and positions of the line profiles within each slice (orange lines), imaged consistently across six staining time points (T0h–T96h). The intensity range for all orthoslices was set between 0 and 40,000 HU. Scale bars = 2 mm. (a) and (b) Reproduced with permission from Camilieri‐Asch et al. (2020 and 2020b), respectively.

**FIGURE 7:**
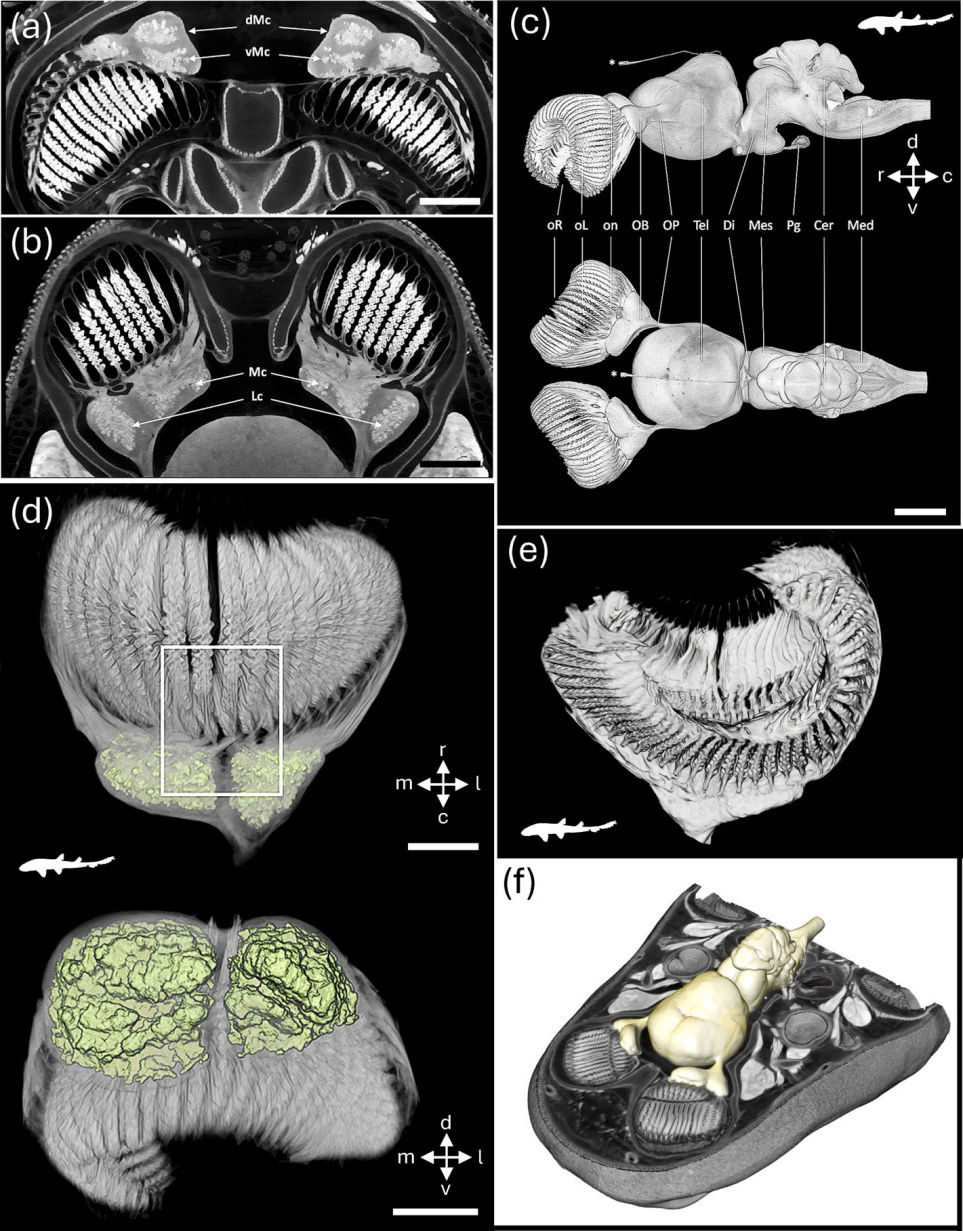
Bioimaging of the nervous system in the bamboo shark. (a, b) Orthoslices of the rostral region of *Chiloscyllium punctatum* showing dorsoventral (a) and frontal (b) slices of the olfactory peripheral nervous system and part of the forebrain, including the olfactory bulbs (OBs) and the anterior portion of the telencephalon. Note the compartmentalization of glomeruli in each OB with two main medial and lateral clusters of glomeruli (a) and the subdivision into dorsal and ventral parts in the posterior part (b). Mc, medial glomerular cluster; Lc, lateral glomerular cluster; dMC, posterior‐dorsal medial glomerular cluster; vMc, posterior‐ventral medial glomerular cluster. Scale bars = 2.5 mm. (c). Lateral view (top) and dorsal view (bottom) of the brain and olfactory peripheral nervous system (PNS) in *C. punctatum*, segmented from μCT images in Avizo. *, pineal gland and nerve; oR, olfactory rosette; oL, olfactory lamella; on, primary olfactory nerve bundle; OB, olfactory bulb; OP, olfactory peduncle; Tel, telencephalon; Di, diencephalon; Mes, mesencephalon; Pg, pituitary gland; Cer, cerebellum; Med, medulla oblongata. Scale bar = 5 mm. c, caudal; d, dorsal; r, rostral; v, ventral. (d) Volume‐rendered olfactory bulb and rosette of *C. punctatum* segmented with the glomerular clusters segmented and labeled in color. The upper image shows the dorsal view of the olfactory bulb and the lateral and medial glomeruli clusters. The lower image is the anteroposterior view of the olfactory bulb and glomeruli clusters (color). m, medial; l, lateral; r, anterior; c, posterior; d, dorsal; v, ventral. Scale bars = 5 mm. (e) Avizo animation of the olfactory rosette revealing the two segmented clusters of glomeruli in the right olfactory bulb of the brownbanded bamboo shark *Chiloscyllium punctatum*, suggesting a potentially somatotopic or zonal organization in the integration of primary inputs in the olfactory system of this species. See Video 3 for the animation. (f) Avizo animation of head revealing the central nervous system (brain) and peipheral sense organs of the brownbanded bamboo shark *Chiloscyllium punctatum*, showing the levels of contrast between and within soft tissues. See Video 4 for the animation. (a‐d) Reproduced with permission from Camilieri‐Asch, Shaw, Yopak, et al. (2020).

**FIGURE 8:**
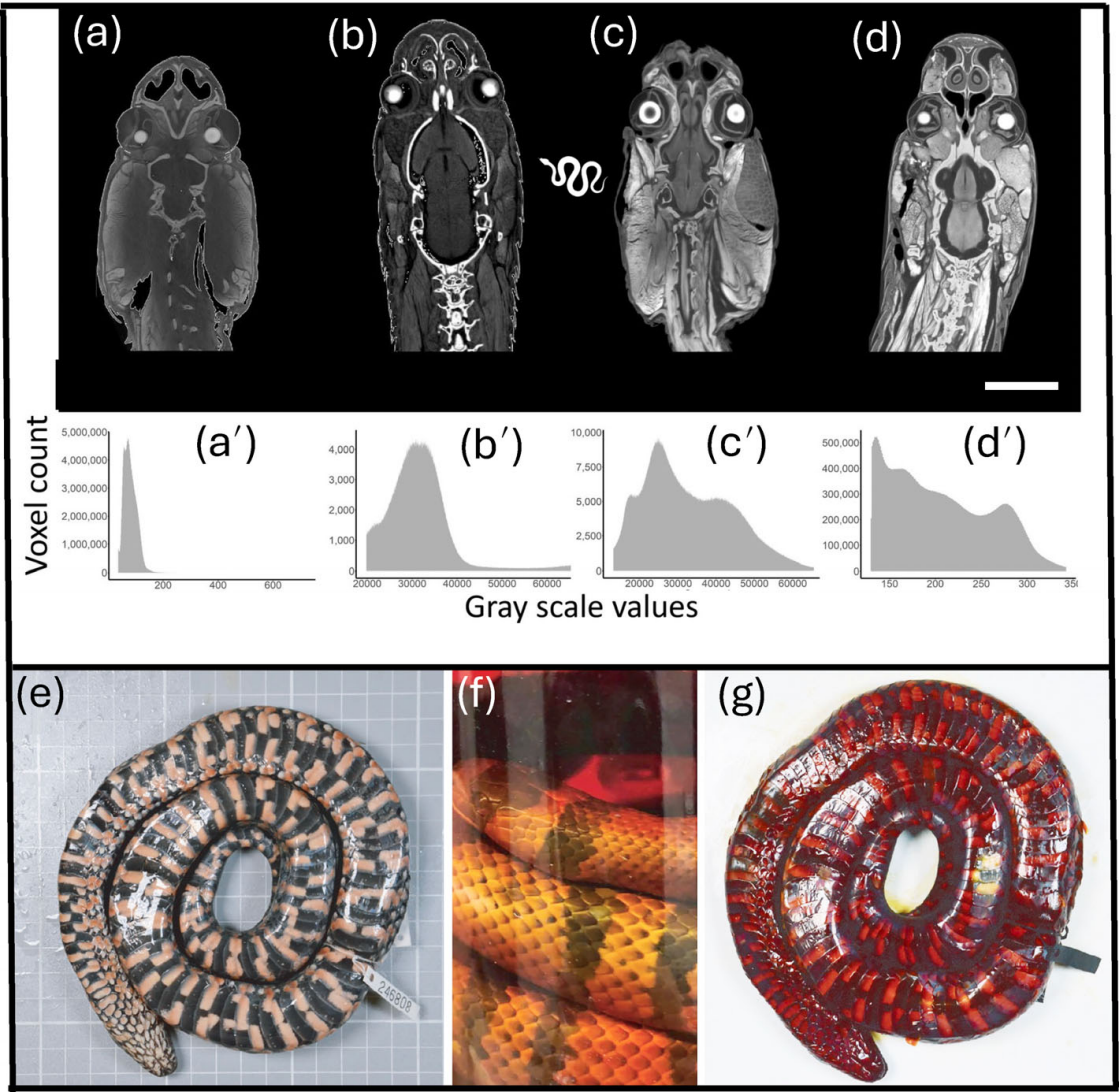
Staining optimization and diffusible iodine‐based contrast‐enhanced computed tomography (diceCT) in snakes. (a–d). Dorsal tomography slices of different species of snakeheads: *Pseutes sulphueus* (a); *Aparallactus capensis* (b); *Bothrops bilineatus (c)*; *Tantilla melanocephala* (d) following staining in 1.25% Lugol's iodine solution between 3 and 11 days. (a'–d'). Corresponding histograms show the distribution of grayscale values for select specimens and thereby a visual indication of the level of iodine staining in preserved snakes. (e) Ventral view of an unstained snake specimen. (f) A specimen immersed in 1.25% Lugol's iodine solution has become partially transparent. The transparent solution indicates incomplete saturation of the specimen and the need for the solution to be replaced with fresh 1.25% Lugol's. (g) Ventral view of the same specimen shown fully stained. Scale bar = 2 cm. Figures all reproduced with permission from Callahan et al. (2021).

Lugol's iodine solution (I_2_KI) is a highly effective agent for rapidly differentiating many types of soft tissues (based on their lipid contents), such as the brain and other aspects of the peripheral and CNSs (Gignac et al., 2016; Gignac & Kley, 2014, 2018; Metscher, 2009). Iodine and its byproducts bind preferentially, but differentially, to lipids present in myelinated nervous tissue and its use has more recently been named diffusible‐iodine contrast‐enhanced CT or diceCT by Gignac et al. (2016). High‐resolution 3D data from a range of neural structures in fishes (both bony and cartilaginous) has been collected using μCT scanning of specimens stained in I_2_KI, for example, brain, cranial nerves, olfactory rosettes, and membranous inner ear structures (Camilieri‐Asch, Caddy, Hubbard, et al., 2020; Camilieri‐Asch, Shaw, Mehnert, et al., 2020; Camilieri‐Asch, Shaw, Yopak, et al., 2020; Chapuis et al., 2023) (Figures 6 and 7). The same technique has been used in reptiles, such as a detailed description of cranial nerves across the ontogeny of alligators (Lessner & Holliday, 2022), the evolution of cerebellar shape in lizards and snakes (Macrì et al., 2019, 2023), and the embryonic brain and CNS development in a parthenogenetic gecko (Griffing et al., 2019). Lugol's iodine solution can also visualize soft‐tissue structures throughout the body, which is useful for describing anatomical structures that are accessories to sensory systems such as the otic‐swim bladder connection in some fishes (Kolmann et al., 2023) or the harderian glands and bifurcated tongue linked to vomerolfaction in snakes and some lizards. Protocols for scanning fishes of varying body shapes (Kolmann et al., 2023) and elongate snakes (Callahan et al., 2021) describe how to optimize Lugol's iodine staining especially for high‐throughput μCT scanning of museum specimens (Figure 8). I_2_KI has been shown to cause minor soft‐tissue shrinkage (Camilieri‐Asch, Shaw, Mehnert, et al., 2020; Vickerton et al., 2013), which can be reduced if using a buffered I_2_KI solution (or “B‐Lugol”) (Dawood et al., 2021). If used at lower concentrations (up to 2.5%), buffered Lugol's iodine solution does not appear to cause excessive tissue shrinkage and any tissue discoloration is reversible via destaining (Callahan et al., 2021; Dawood et al., 2021; Descamps et al., 2014; Gignac et al., 2016; Kolmann et al., 2023), allowing for certain histological characterization techniques (Gignac et al., 2021).

Another popular contrast agent is PTA, which binds heavily to various proteins and connective tissue (Metscher, 2009) and has also been used to stain sensory organs, like the sensory epithelia of fish inner ears (Schulz‐Mirbach et al., 2013). Similar to PTA is PMA (phosphomolybdic acid), which successfully stains the chemosensory system (vomeronasal organs) in different lizard species (Baeckens et al., 2017) and the olfactory rosettes in three species of blenny (Ajavon & Simonitis, 2022). Both PTA and PMA increase the density of soft tissues and appear to provide reduced tissue shrinkage during staining, compared to the I_2_KI solution (Descamps et al., 2014; Greef et al., 2015). Although PTA and PMA cannot be removed from the specimen once stained, they do not cause obvious discoloration of the tissue like the I_2_KI solution (Figure 8). Furthermore, PTA staining time differs largely between fishes and reptiles, with reptile skin almost entirely preventing stain uptake through the scales, where skinning or incisions in the body cavity are required (pers. comm. Jaimi Gray and Qamariya Nasrullah).

The storage solution used between fixation and staining times is also important to consider based on the sensory system of interest or the staining agent selected. If choosing a fixative storage solution, avoiding ethanol‐based solutions, which tend to dissolve lipids, is paramount when using iodine‐based staining agents, to ensure that stain uptake is maintained in the nervous tissues of interest (Gignac et al., 2016). Staining time can vary depending on the fixative of choice, and time spent in the fixation solution. If choosing a buffer option, then using a cacodylate buffer instead of a phosphate buffer might be preferred when staining with PTA and studying the inner ears, to avoid crystal formation during the fixation process near or on the surface of otoliths (Schulz‐Mirbach et al., 2013).

### 4.1 Optimizing contrast

Optimizing contrast in CT‐imaging of the nervous system is primarily achieved by optimizing staining concentration and staining time. Under‐ and over‐stained samples produce poor contrast among the different types of soft tissues, providing poor differentiation of sense organs and the CNS, thus creating difficulty for subsequent segmentation of regions of interest (Figure 8). Although over‐staining can sometimes be mitigated using an aluminum filter during scanning, optimal staining times will ultimately depend on the circumference of the widest part of the specimen, as well as any particular morphological feature of the species, for example, the presence of a shell in turtles. Preliminary, fast (scout) scans taken at fixed time stamps can assist in constructing a protocol for optimizing the contrast agent concentration and the staining time for any given species (Camilieri‐Asch, Shaw, Mehnert, et al., 2020; Callahan et al., 2021, Figures 5 and 6).

The compressed body shape of some species of fishes and reptiles provides a larger surface area‐to‐volume ratio and typically offers improved stain uptake. Extensive squamation of the skin and surface structures of fishes and reptiles, such as the tooth‐like denticles of most cartilaginous fishes, the large and thick scales of many early (non‐actinopterygian) fishes, and the osteoderms of many reptiles will also limit diffusion (Kolmann et al., 2023). In this case, incisions in the skin can increase stain uptake inside the body cavity. Note that these skin incisions should not be made too close to the soft tissues of interest, as they can entrap air bubbles, creating motion blurring and image artifacts, thus reducing scan quality. Turtles are particularly challenging for immersive staining techniques given their large shells formed by bony plates that create a barrier to diffusion. A new protocol details injecting Lugol's solution using a hypodermic needle directly under the shell and into the body cavity, resulting in detailed neuroanatomy (Gray et al., 2024). Selectively perfusable iodine‐based contrast‐enhanced CT (spiceCT) is a technique that rapidly stains freshly deceased specimens by injecting Lugol's iodine solution into the arterial system (Witmer, 2018). Although a rapid and straightforward technique, it fails to stain the brain, thereby highlighting the challenges in using contrast agents to stain the CNS.

During iodine staining of intact animals, the brain is often the last structure to adequately uptake stain because it is buried deep within the cranium, requiring diffusion throughout all surrounding tissues. One approach to optimizing staining time for neurological tissues is to increase the concentration of the staining solution and/or dissect the brain (or other regions of interest) and remove it from the head, rather than staining the whole specimen, although this approach negates the non‐destructive nature of the methodology. Less destructive approaches to maintaining brain integrity include submerging the intact head (with or without the skin) into the staining solution, as the stains can penetrate the endocast faster and more directly by diffusing along the spinal cord. Alternatively, if the regions of interest are peripheral end organs with large surface areas (e.g., olfactory rosettes and heat pits) or downstream branches of cranial nerves, then “understaining” specimens and scanning before the stain has reached the brain may provide optimal contrast for those areas. Overstaining can also be reversed, where a stained specimen can be placed in an ethanol or water bath after staining and prior to scanning. This helps wash away unbound iodine and reduces the contrast. Finally, regularly replacing the staining solution to maintain a high diffusion gradient is an ideal strategy to optimize staining time (Camilieri‐Asch, Shaw, Mehnert, et al., 2020; Gignac et al., 2016). While stain uptake is visible with iodine‐based agents like I_2_KI (i.e., lightly colored solution will indicate that it can be changed, Figure 8), PTA (typically 1%–5%) and PMA remain transparent and are therefore harder to monitor with respect to infiltration times, although changes in pH as a result of a reduction in concentration of the staining solution can signify uptake of the stain to the tissues.

There are potential risks to specimens using I_2_KI. Iodine staining causes discoloration of specimens but this is largely reversible through immersion in ethanol (25%–70%) or in a sodium thiosulfate solution (<10%). However, preliminary studies show that sodium thiosulfate may cause decalcification of bone (Mataic & Bastani, 2006), so it is recommended to use very low concentrations to safely destain specimens (<1%, Jaimi Gray, pers. comm). I_2_KI has been shown to cause soft‐tissue shrinkage especially at very high concentrations (20%–70% in mice) (Vickerton et al., 2013). However, a study that measured brain and eye shrinkage in bats found no significant effects of Lugol's staining at 2.5% concentration over 4 weeks (Yohe et al., 2018). Furthermore, any potential shrinkage can be effectively mitigated using a buffered I_2_KI solution (“B‐Lugol”) to keep the solution isotonic (Dawood et al., 2021). Demineralization can also occur at moderate concentrations (3%–6%) and/or longer staining durations in Lugol's solution (Early et al., 2020). Therefore, the lowest possible concentration should be used (1%–2.5%) in a buffered Lugol's solution. Further studies are needed to assess the potential differential rates of tissue shrinkage between central and peripheral nervous systems among fishes and reptiles.

### 4.2 Optimizing resolution

Optimizing resolution in μCT imaging is crucial for obtaining high‐quality, detailed images that can reveal the fine structures of the central and peripheral nervous systems. A high pixel density and small pixel size on the detector will allow for higher spatial resolution. Adjusting the source‐to‐object and the object‐to‐detector distances will also significantly impact the magnification and resolution. For instance, reducing the source‐to‐object or increasing the object‐to‐detector distances can enhance magnification, thereby improving the resolution. However, this might reduce the field of view, and therefore mask some of the region(s) of interest. Another critical issue is the position in which the body is fixed. For example, a coiled snake versus a hairpin‐shaped snake can have a substantial effect on image quality. This is for two reasons. Firstly, how close the specimen is to the source/detector is also dependent on the animal's size and configuration, which can limit the resolution, especially for full‐body scans. Secondly, these same two factors can also affect the penetration of the X‐rays and have an impact on image quality and the SNR. Therefore, a balance must be struck based on the image requirements and generating enough powerful X‐rays that can penetrate dense, iodine‐imbued tissues, while minimizing visual noise (Gignac et al., 2016; Neu & Genin, 2014). Optimizing the scanning parameters, including the X‐ray voltage, current, and exposure time, is paramount for obtaining high‐quality images (Gignac et al., 2016). Finally, the sample should be well stabilized and positioned with the region of interest (i.e., the head) centered to minimize motion artifacts. Advanced reconstruction algorithms can also enhance the effective resolution by reducing noise and artifacts. The use of phantom scans (air and water) can be used to calibrate the reconstruction of raw scan files (Camilieri‐Asch, Shaw, Mehnert, et al., 2020, Figure 6b). This can be facilitated by including standards, for example, an aluminum rod or some other material of known density, in with the tissue to ensure scan data is consistently calibrated, thereby enabling multiple scans to be stitched together to maximize resolution. Post‐processing techniques, such as deconvolution and filtering, will also improve the final image quality and resolution (Clark et al., 2005), for example, Enhance Local Contrast (Contrast Limited Adaptive Histogram Equalization, CLAHE) plugin in Fiji imaging software (Schindelin et al., 2012).

### 4.3 Phase contrast micro‐computed tomography

Conventional CT systems rely on the attenuation of X‐rays to improve contrast. In addition to being absorbed, the X‐ray wavefront interacts with the sample by being phase shifted. This phase shift is caused by the speed changes dependent on the density and composition of the material in the sample. The phase shift is more sensitive to variations in tissue density and composition than X‐ray absorption. Therefore, X‐ray phase‐contrast imaging offers an alternative method to enhance soft tissue contrast (Arhatari et al., 2021). Several established methods providing X‐ray phase‐contrast exist, the most simple being propagation‐based imaging (Paganin & Pelliccia, 2021). This type of imaging requires either a dedicated synchrotron light source or a highly specialized laboratory‐based light source with required scan times often exceeding several hours to days.

### 4.4 Synchrotron‐based computed tomography

A synchrotron light is produced when high‐energy electrons travel in a circular orbit inside the synchrotron tunnels assisted by strong magnetic fields. Synchrotron radiation is extremely bright and intense, compared to conventional X‐ray sources. Together with high‐resolution detector systems, this high‐intensity beam allows for rapid imaging with high temporal and spatial resolution, enabling dynamic studies and reducing scan time.

Synchrotron‐based computed tomography has proven to be one of the most powerful X‐ray imaging techniques with enough contrast to detect microstructural components, such as individual neurons (Hwu et al., 2017). Synchrotron‐μCT has notably enabled the cellular characterization of the zebrafish and medaka brain and various species of reptiles, allowing brain nuclei to be computationally segmented and assigned to brain regions (Broekhoven & du Plessis, 2018; Ding et al., 2019; Weinhardt et al., 2018). Several initiatives are aiming to fully map all the neurons and their connections in the human brain and other model animal brains, that is, *Drosophila* spp. and the house mouse *Mus musculus* (Chin et al., 2020; Stampfl et al., 2023).

There are more than 50 synchrotron facilities (operational or under construction) around the world, all facilitating research using X‐ray imaging. The peripheral and CNS of several species of cartilaginous and bony fishes (Figure 2b), and reptiles, have been imaged by our Group at the Imaging and Medical beam line (IMBL) of the Australian Synchrotron (Arhatari et al., 2021).

### 4.5 Other advances in micro‐computed tomography

Over the past two decades, there have been significant advances in μCT technology and dramatic increases in spatial and temporal resolution, through a combination of improved energy sources (synchrotron and laboratory tube sources), detectors, and reconstruction algorithms, notably making novel contributions in the field of neuroscience (Rodrigues et al., 2021). New imaging methodologies continue to emerge and advance (Withers et al., 2021). For instance, dual‐energy μCT, also known as spectral μCT, uses two different X‐ray energy levels (voltages) in sequence or simultaneously to acquire imaging data (McCollough et al., 2015). This technology allows for the differentiation of materials within the specimen based on their atomic number, thereby enhancing the contrast and detail observed within the images. Thus far, dual‐energy CT has mostly been used in assessing brain diseases and injuries in a human clinical setting (Xu et al., 2024).

Other novel techniques include cryogenic contrast‐enhanced μCT and X‐ray fluorescence μCT. With cryogenic contrast‐enhanced μCT, a tissue sample is stained and then frozen at an optimal rate to enable 3D visualization and structural analysis of individual tissue components, such as muscle and collagen fibers (Maes et al., 2022). This method overcomes the current limitations of contrast‐enhanced CT (i.e., shrinkage and low contrast) without the need for more specialized techniques, such as synchrotron‐based phase contrast, and may present an opportunity to observe the nervous system of animals such as fishes and reptiles. X‐ray fluorescence μCT uses a scanning probe that detects the fluorescence of a given chemical compound, similar to that of a confocal microscope. In combination with intense synchrotron‐based X‐rays, this method achieves nanometer spatial resolution of intracellular probe elements in brain tissue (Collingwood & Adams, 2017). As such, it has proven to be a very sensitive technique to investigate the neurotoxicity of heavy metals in the animal brain (Webb et al., 2022).

## 5 USES OF MRI AND μCT IN COMPARATIVE NEUROANATOMY

Magnetic resonance imaging has long been a widely used tool for comparative neuroanatomy, although its utility in non‐mammalian and non‐model species has been comparatively unexplored until recently. This includes the ability to characterize neuroanatomy in unique taxa (Anderson et al., 2000; Jiménez et al., 2024; Marino et al., 2003; Oelschlager et al., 2008; Schmidt et al., 2009; Yopak & Frank, 2009), explore brain to endocast ratios across ontogeny (Jirak & Janacek, 2017), develop high‐resolution 3D brain atlases (Billings et al., 2020; Foss et al., 2022; Hoops et al., 2021; Hoops, Vidal‐Garcia, et al., 2017; Poirier et al., 2008; Ullmann, Cowin, et al., 2010c), explore the capacity of neuronal current‐induced MRI (Luo et al., 2009), and study embryonic development and neuroplasticity *in vivo* (Hogers et al., 2009; Van der Linden et al., 2004; Van Meir et al., 2006). It has also been applied to assessments of barotrauma (Rogers et al., 2008) and investigations of highly specialized internal structures in fishes (Chakrabarty et al., 2011; Forbes et al., 2006; Graham et al., 2014; Scadeng et al., 2020; Sepulveda et al., 2007). MRI has been instrumental in the generation of online databases that disseminate MR scans (Berquist et al., 2012; Ziegler et al., 2014), where new comparative questions can be asked of digital data (Toga, 2002).

Magnetic resonance imaging includes both anatomical (or structural) and functional applications. Introduced only within the last four decades (Biswal et al., 2010), functional MRI (fMRI) aims to assess variation in metabolic activity in the brain at various time points. This includes changes in the ratio of oxygenated to deoxygenated hemoglobin (i.e., blood‐oxygen‐level‐dependent (BOLD) signals), or changes in cerebral blood flow or volume (CBV). The application of fMRI is underutilized in studies of fishes and reptiles, due to numerous considerations and challenges (see review by Van der Linden et al., 2007). Despite the challenges, studies do exist in both taxa. For example, van den Burg et al. (2005) utilized the BOLD signal and CBV in various hypothalamic nuclei and pituitary to explore thermal stress in a cyprinid (the common carp *Cyprinus carpio*), and later characterize the sensory‐motor pathway in response to a drop in temperature (van den Burg et al., 2006). More recently, BOLD signal changes were measured in the Nile crocodile, *Crocodylus niloticus*. This study documented BOLD increases and decreases in spatially and functionally segregated forebrain regions in response to visual or auditory stimuli, demonstrating conserved sensory processing networks in sauropsids, as well as the potential viability of fMRI for poikilotherms (Behroozi et al., 2018). However, outside of these studies, much more work is required to optimize the application of fMRI and determine its utility in assessing neural activity in fishes and reptiles.

In contrast to fMRI, structural or anatomical MRI has gained momentum in recent years to investigate internal anatomy, including peripheral and CNS characterization in fishes and reptiles. While early scans of specimens could only enable the identification of major body structures *in situ* (Blackband & Stoskopf, 1990; Cloutier et al., 1988; Waller et al., 1994), advances in these methods have greatly enhanced our ability to acquire very high‐resolution images with high contrast in nervous system tissue, including the ventricular system (Jiménez et al., 2024). Anatomical MRI technology and generation of 3D data has now been used to characterize inter‐ and intraspecific variation in peripheral structures and the brain in a range of studies (DePasquale et al., 2016; Hoops et al., 2018; Hoops, Ullmann, et al., 2017; Jiménez et al., 2024; Peele et al., 2023; Triki et al., 2021; Ullmann, Cowin, et al., 2010c; Ullmann, Cowin, & Collin, 2010b). For example, MRI was used to characterize ocular and retinal specializations in the triplefin blenny *Tripterygion delaisi* (Fritsch et al., 2017, Figure 4), and ocular diverticula in the tubular eyes of deep‐sea fishes (Opisthoproctidae, Wagner et al., 2022) and to measure both eye and lens size in psammophilic lizards (Canei et al., 2020). With respect to other sensory modalities, MRI and/or μCT have been used to investigate the adaptive responses of inner ear bones (otoliths) to altered gravity in toadfishes (Boyle, 2021), paratympanic sinuses in the alligator Tahara and Larsson (2022), and the comparative volumes of the olfactory rosette in both cartilaginous and bony fishes (Camilieri‐Asch, Caddy, Hubbard, et al., 2020). Bandoh et al. (2011) have also applied BOLD fMRI to investigate odor information processing in the olfactory bulb and telencephalon in the brain of lacustrine sockeye salmon (*Oncorhynchus nerka*), while Lessner et al. (2023) have examined the ecomorphological patterns in trigeminal canal branching among sauropsids.

With respect to studies of comparative neuroanatomy of the brain, Triki et al. (2021) scanned the brains of 20 adult female cleaner wrasse *Labroides dimidiatus* and found correlations between population densities and forebrain size. Following rearing in enriched versus barren environments and subsequent anxiety and learning assays, MR scans of *Danio rerio* brains revealed larger brains in individuals reared in complex environments alone (DePasquale et al., 2016). MR imaging can also enable the examination of nervous system tissue in rare or endangered specimens such as large‐bodied sharks (Yopak & Frank, 2009), the Nile crocodile (Billings et al., 2020), and the rhinoceros iguana (González et al., 2023). The incorporation of MR data of rare or vulnerable species into large, comparative datasets of species with known morphological or behavioral specializations can help predict their ecological and conservation requirements. For example, the inclusion of volumetric data from MRI on major brain regions in the whale shark *Rhincodon typus* (Yopak & Frank, 2009, Figure 3), the Greenland shark *Somniosus microcephalus*, and the Pacific sleeper shark *Somniosus pacificus* (Yopak et al., 2019) in a large comparative dataset from cartilaginous fishes shows close correlations between neuroanatomical specializations and ecology (see Yopak, 2012, 2022 for reviews). Such large comparative MR data are not yet available for reptiles, but will likely yield similar insights into behavioral and ecological variation (Jiménez et al., 2024).

**FIGURE 9:**
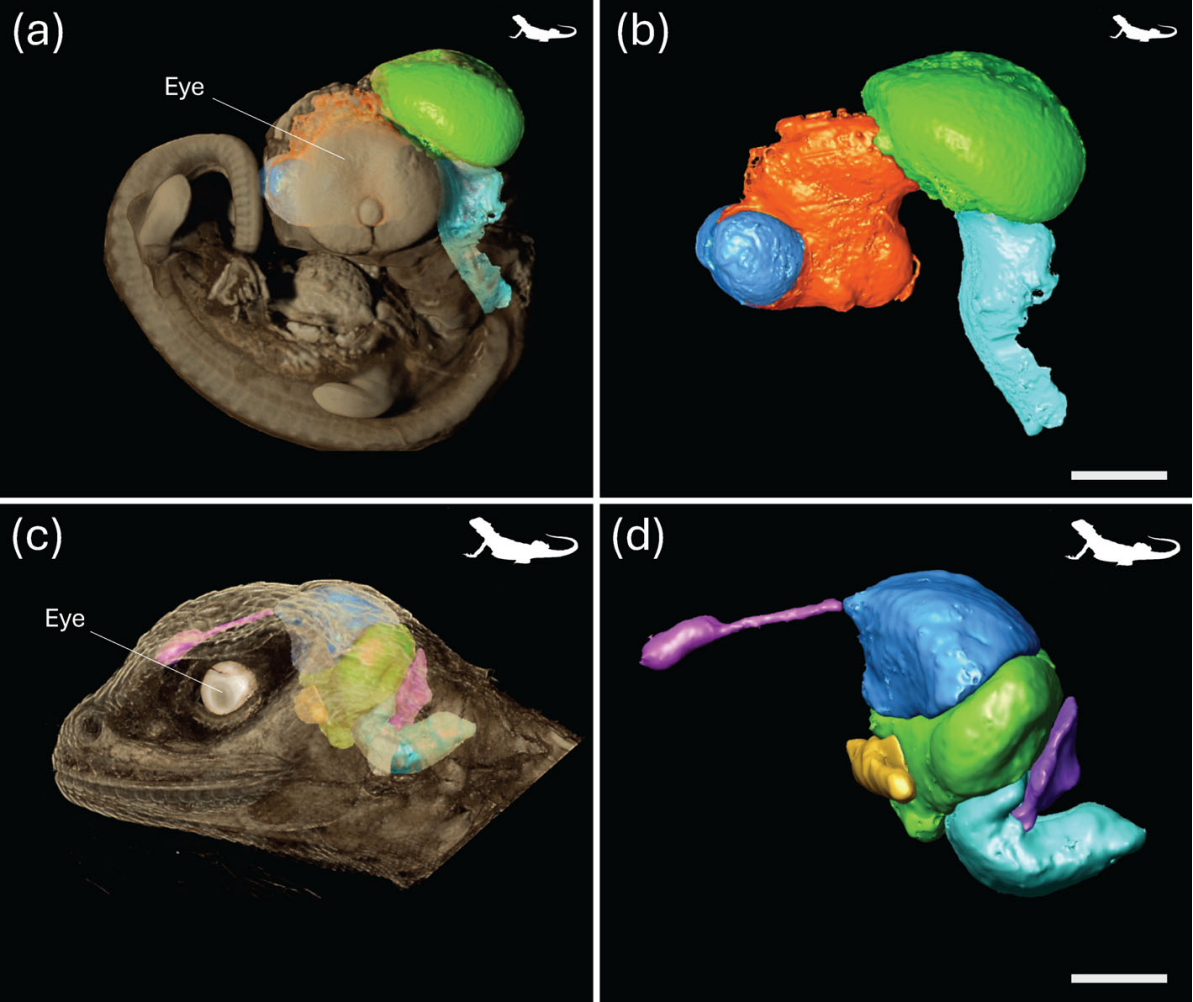
Using diceCT to understand the growing embryonic brain in the bearded dragon *Pogona vitticeps*. (a) 3D render of a 6‐day‐old embryonic brain showing the early developmental stages of the telencephalon (blue), diencephalon (orange), mesencephalon (green), and rhombencephalon (aqua). (b) A 3D reconstruction of the 6‐day‐old embryo showing the enlarged eyes and the position of the brain within the body. Scale bar = 1 mm. (c) A 3D reconstruction of the 70‐day‐old embryo head showing the more well‐portioned eye and the position of the brain within the head. (d). 3D render of a 70‐day‐old embryonic brain showing the late‐developmental stages of the olfactory bulbs and stalk (pink), telencephalon (blue), optic nerve (yellow), mesencephalon (green), cerebellum (purple), and rhombencephalon (aqua). Scale bar = 1 cm. All reconstructions were performed using diceCT scanning and Dragonfly software.

Integrating MRI and μCT with traditional neurological techniques has advanced the development of brain atlases, providing accurate anatomical references and volumetric information in 3D. MR‐based brain (or brain subregion) atlases now exist for a range of reptiles, including the garter snake *Thamnophis sirtalis* (Anderson et al., 2000), tawny dragon *C. decresii* (Hoops et al., 2018, 2021, Figure 5a–c), Nile crocodile *C. niloticus* (Billings et al., 2020), and bearded dragon *Pogona vitticeps* (Foss et al., 2022, Figure 9), with resolution improving in the last several decades as more technological advances have become available. Most recently, an atlas for the prosencephalon, acquired from MR data, was produced for the pond slider *Trachemys scripta*, central bearded dragon *P. vitticeps*, and ball python *Python regius* (Jiménez et al., 2024). Similarly, a range of atlases has been produced in several bony fishes, including the zebrafish *D. rerio* (Ullmann, Cowin, et al., 2010c), barramundi *L. calcarifer* (Ullmann, Cowin, & Collin, 2010b), Mozambique tilapia *Oreochromis mossambicus* (Simoes et al., 2012), and African lungfish *Protopterus annectens* (Lozano et al., 2024). Studies have even applied super‐resolution track density imaging to *D. rerio*, resolving fiber tracts and laminae within the optic tectum (Ullmann et al., 2015, Figure 1c). While the majority of these atlases have acquired anatomical data on fixed tissue, recent studies have developed protocols to acquire anatomical data *in vivo*. For example, Clark et al. (2022) produced a limited MR‐based brain atlas from *in vivo* imaging of three teleosts, the channel catfish *Ictalurus punctatus*, the koi *Cyprinus rubrofuscus*, and grass carp *Ctenopharyngodon idella*. Foss et al. (2022) scanned seven, live *P. vitticeps* on a 3 T, developing the first *in vivo* MR brain atlas in a reptile. This greatly opens the door to improve diagnostic capabilities in veterinary medicine and the exotic pet trade (Głodek et al., 2016; Mathes et al., 2017; Ruiz‐Fernández et al., 2020; Schrenk et al., 2022), in addition to enabling longitudinal analyses in a range of species to quantify effects of environment and temperature on brain growth using both MRI and μCT (Figure 9).

The 3D nature of MR and μCT data can also allow for the application of automated shape analysis techniques, where variation in surface complexity of neural structures can be mathematically modeled and compared across species in both fishes (Yopak et al., 2016) and reptiles (Macrì et al., 2019). Similar advances are now being explored using three‐dimensional (3D) bioimaging, geometric morphometrics, and finite element analysis to interrogate structure–function relationships in the inner ears of elasmobranchs (Chapuis et al., 2023; Sauer et al., 2023). Some studies in birds and reptiles have even combined MRI and μCT for quantitative analyses of the brain utilizing an interactive Fakir probe cross‐referenced with an automated CT protocol to calculate the volume and surface area of the brain and endoneurocranial space (González et al., 2023; Jirak et al., 2015).

### 5.1 Segmentation of target tissues

**FIGURE 10:**
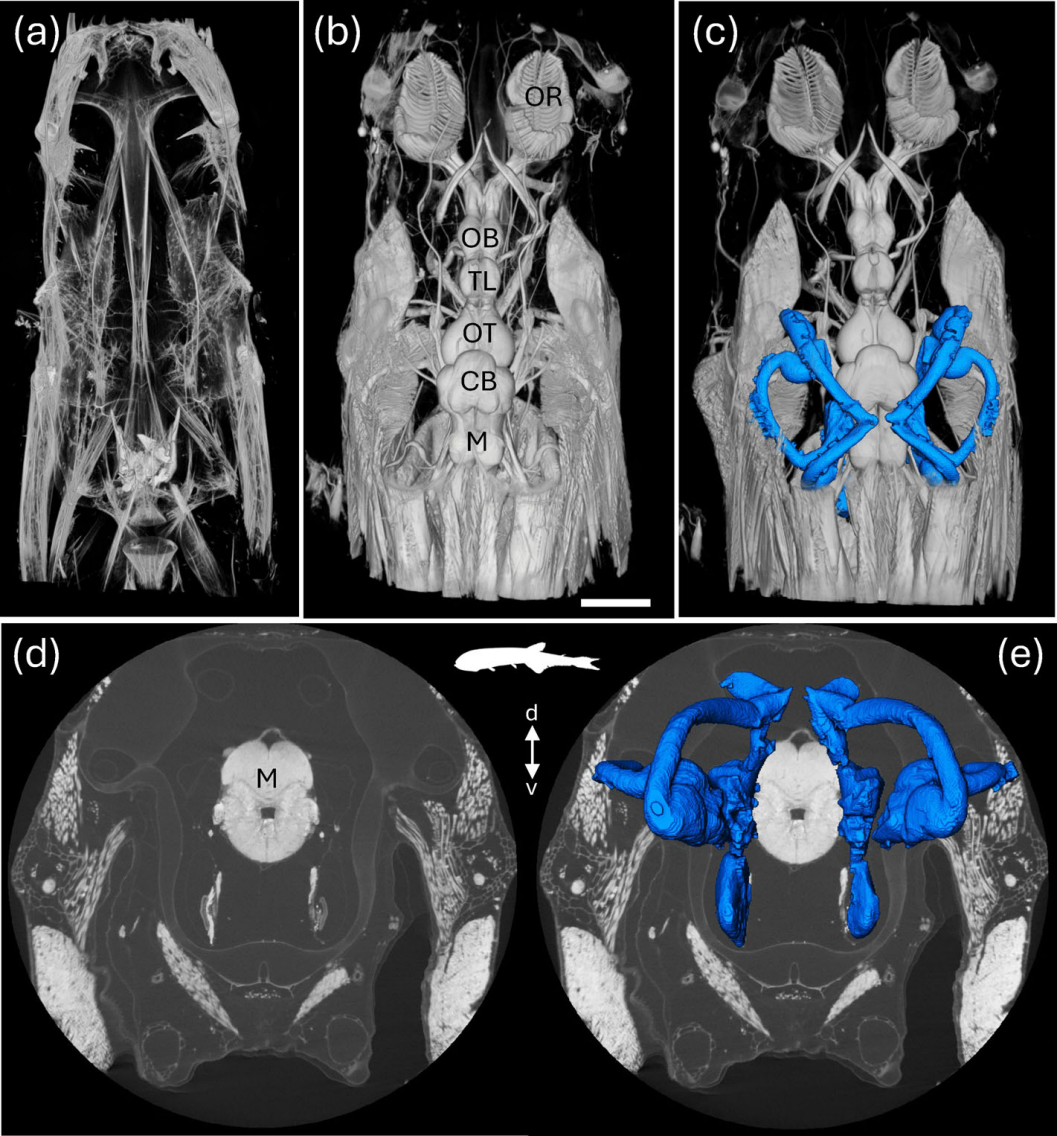
Bioimaging in the deep‐sea bristlemouth *Sigmops elongatus* using diceCT. (a). μCT scans without contrast enhancement show only the hard components of the skeleton. (b) Scan taken 72 h later following staining with Lugol's iodine solution. The specimens were scanned using an X‐ray micro‐computed tomography system (μCT) (Versa 520 XRM, Zeiss, Pleasanton, CA) running Scout and Scan software (v11.1.5707.17179) at the Centre of Microscopy, Characterization and Analysis (CMCA) at The University of Western Australia. Segmented membranous labyrinth and inner ear maculae are shown in blue (c, e) following the reconstruction of 2D image slices using XMReconstructor and importation into the software package Avizo 9.0 (d). CB, cerebellum; M, medulla oblongata; OB, olfactory bulbs; OR, olfactory rosette; OT, optic tectum; TL, telencephalon. D, dorsal; v, ventral. Scale bar = 2 cm. Images courtesy of J. Kreig, L. Chapuis, and S. P. Collin.

**FIGURE 11:**
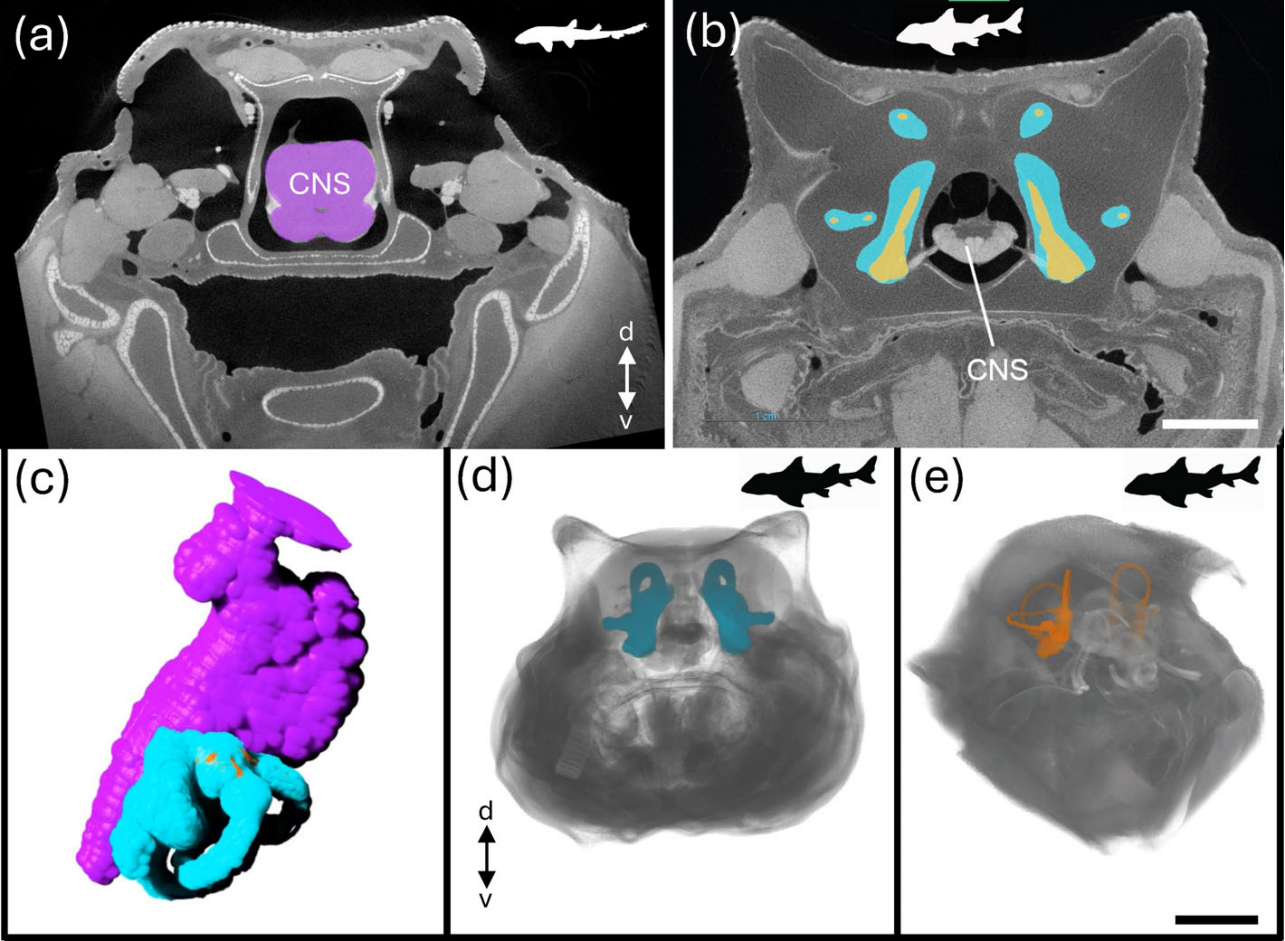
Bioimaging of the brain and inner ears in the juvenile epaulette shark *Hemiscyllium ocellatum* (a, c) and the juvenile Port Jackson shark *Heterodontus portusjacksoni* (b, d, e). (a) Axial X‐ray μCT coronal image stack through the head of *H. ocellatum* at the level of the midbrain. See Video 5 for the video animation. (b) Axial X‐ray μCT coronal image stack through the head of *H. portusjacksoni* at the level of the hindbrain. Segmentation was semi‐automatically performed using Dragonfly software. Purple depicts brain tissue of the central nervous system (CNS), while the blue and yellow/orange areas depict the outlines of the cartilaginous and membranous labyrinths, respectively. Scale bar = 2 cm. (c) Animated segmentation of the brain and both labyrinths in *H. ocellatum*. See Video 6 for the video animation. (d, e). 3D views of the fully reconstructed labyrinths of the inner ear of *H. portusjacksoni* within the head in frontal (d) and dorso‐frontal (e) views. Scale bar = 3 cm. Specimens were fixed in 4% paraformaldehyde in 0.1M phosphate buffer, stained with a 1.25% I_2_KI (Lugol's iodine) solution, and scanned with a Phoenix Nanotom M μCT instrument at the Melbourne TrACEES platform. d, dorsal; v, ventral.

**FIGURE 12:**
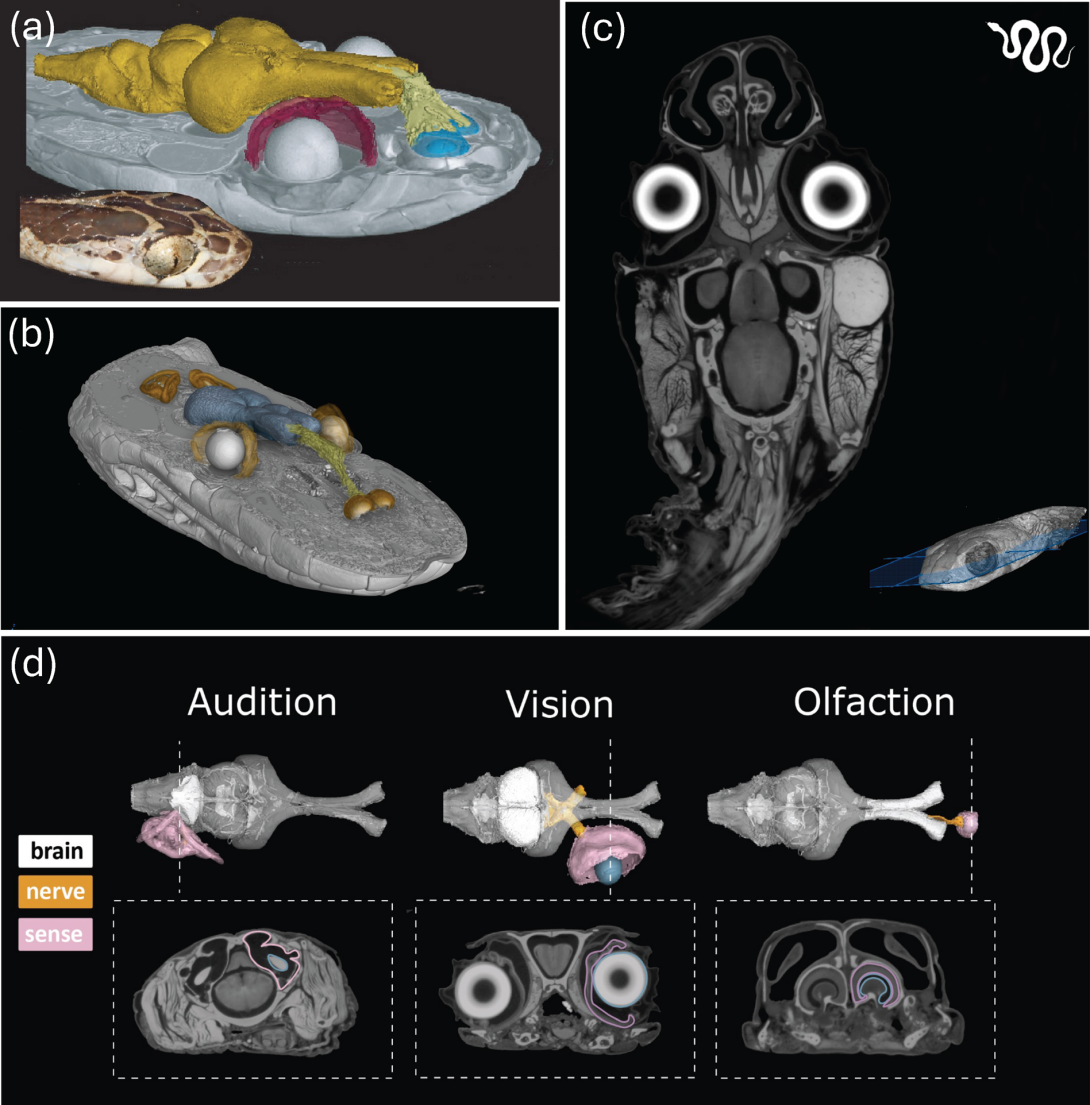
Bioimaging the nervous system of snakes for integrated analysis of multiple sensory systems, allows for the quantification of the relative size of the peripheral sense organs, their cranial nerves and terminal sensory brain regions within the central nervous system (CNS). (a) The nervous system of the blunthead tree snake *Imantodes cenchoa in situ* created by combining an unstained μCT scan (skull segmentation) and iodine stained μCT scan (soft‐tissue segmentation) showing various sensory systems: the vomeronasal (light yellow and blue regions of the olfactory bulb) and visual (retina in red) systems (CNS is dark yellow), next to the head of the live snake. (b) A similar approach to (a) for the Stimson's python *Antaresia stimsoni* with the CNS shown in blue and the vomeronasal tissue and olfactory bulbs shown in light yellow and orange, respectively. The lens (white) and the surrounding retina (light brown) and the segmented inner ears (orange) of the auditory system are also shown. (c) Animation of dorso‐lateral “fly‐through” of a contrast‐enhanced μCT scan of the blunthead tree snake *Imantodes cenchoa* that was stained using 1.25% Lugol's iodine solution and segmented and animated using VG Studio MAX software (Volume Graphics, Heidelberg, Germany) at the University of Michigan Museum of Zoology. See Video 7 for the animation. (d). Breakdown of a neuroanatomical method, which can be used to quantify the volume of a segmented inner ear (Audition), the retina and lens (Vision), and vomeronasal organ and olfactory bulbs involved in the tongue flicking sense (Olfaction) through a series of orthoslices using μCT (lower panels). The upper panels show the fully segmented sense organs, corresponding cranial nerves, and the central nervous system (in dorsal view). (a, b) Reproduced with permission from Callahan et al. (2021). Specimen numbers are UMMZ190766 (b) and UMMZ246810 (d).

The collection of many hundreds or thousands of images through μCT and MRI scanning can lead to gigabytes of high‐resolution datasets per specimen. To reconstruct the anatomy of the peripheral or CNSs within each specimen, the image data has to be labeled according to the desired level of detail, a process called segmentation. Each voxel (i.e., data point on a 3D grid) of interest is assigned a label as belonging to a specific structure, or region of interest. Many open‐source (e.g., 3D Slicer, Drishti, ITK‐SNAP, and Dragonfly), as well as commercial (Amira/Avizo, VGStudio) computer software programs perform this post‐processing, including the segmentation of large 3D datasets. Segmentation can be performed semi‐automatically when the image SNR and the contrast between the structures to be labeled is high. However, for complex biological structures such as the nervous system, that overlap and intersect with many other anatomical features, segmentation is often a manual process requiring hours or days of labor and often expertise in neural and anatomical biology. This is particularly true for the segmentation of both the bony (or cartilaginous in the case of chondrichthyans) and membranous labyrinths of the inner ear of fishes, for example, in the deep‐sea bristlemouth *Sigmops elongatus* (Figure 10), the epaulette shark, *Hemiscyllium ocellatum* (Figure 11a,c) and the Port Jackson shark, *Heterodontus portusjacksoni* (Figure 11b,d,e) and reptiles, for example, the Stimson's python *Antaresia stimsoni* and the blunthead tree snake *Imantodes cenchoa* (Figure 12). However, this approach can be used to accurately quantify the size of sense organs (ears, eyes, and vomeronasal and olfactory organs) to make broader comparisons with their corresponding sensory brain regions (medulla, optic tectum, and olfactory bulbs) and overall brain size within and across species (Figure 12), thereby constituting an anatomical proxy for the relative importance of several sensory modalities.

Deep learning is currently revolutionizing image de‐noising and segmentation in terms of efficacy, accuracy, and speed (Jonsson, 2023). These innovative approaches are currently being used to post‐process images of invertebrates (Lösel et al., 2023; Toulkeridou et al., 2023), but automated segmentation in fishes and reptiles has not yet received much attention. Some promising open‐source platforms, that is, Biomedisa (Lösel et al., 2020, 2023), allow semi‐automatic segmentation of large biological volumetric images, like the ones collected from μCT, and enable deep learning processes for fully automated segmentation. A number of software tools are being used to process 3D data in other vertebrates (mice to humans) but could equally be applied to fishes and reptiles as long as data is acquired to train the algorithms. These include 3D slicer (grow from seeds tool and the extensions MEMOS and Total Segmentator), VG Studio Max (paint and segment tool), and Dragonfly (Deep Learning Solution and Infinite toolboxes) (Dragonfly, 2022). Given the sheer diversity of the sense organs and the brain across the diversity of sensory modalities in fishes and reptiles, fundamental neurobiological research will still be needed to adequately train current machine learning capabilities. Such advances would revolutionize the field of anatomical neurobiology, resulting in big data generation akin to next‐generation DNA sequencing, which will likely bring similar challenges in data management and analysis (Callahan et al., 2021).

## 6 CONCLUSIONS

In conclusion, advances in staining techniques and scanning technologies to enhance the visualization of soft (nervous) tissues *in situ* using MRI, μCT, and super‐resolution track density imaging continue to improve our ability to understand the neuroanatomical diversity of the sense organs and brain and ultimately the sensory abilities of fishes and reptiles. Species‐specific optimization of the protocols for Lugol's iodine and other contrast agents such as PTA and PMA appears to be crucial to preserve the delicate balance between contrast enhancement and tissue integrity and preservation. The adoption of preliminary fast scans as a means of monitoring and refining staining protocols to ensure that the concentration and duration of staining are tailored to each species' requirements is also critical. Together with the advances in software packages for post‐processing of 3D high‐resolution datasets for segmentation and quantification, geometric morphometrics, and finite elemental analyses, *in situ* imaging will remain an important tool to explore the neuroanatomical diversity of fishes and reptiles, and understand the correlations between brain, sensory organs, ecology, and phylogeny.

## AUTHOR CONTRIBUTIONS

**Shaun P. Collin:** Conceptualization; formal analysis; funding acquisition; investigation; methodology; project administration; resources; supervision; visualization; writing – original draft. **Kara E. Yopak:** Data curation; formal analysis; methodology; software; validation; visualization; writing – review and editing. **Jenna M. Crowe‐Riddell:** Data curation; formal analysis; methodology; software; validation; visualization; writing – review and editing. **Victoria Camilieri‐Asch:** Data curation; formal analysis; methodology; software; validation; visualization; writing – review and editing. **Caroline C. Kerr:** Data curation; methodology; software; visualization; writing – review and editing. **Hope Robins:** Formal analysis; methodology; visualization. **Myoung Hoon Ha:** Formal analysis; methodology; software; visualization. **Annalise Ceddia:** Formal analysis; methodology; visualization. **Travis L. Dutka:** Methodology; resources; supervision. **Lucille Chapuis:** Data curation; formal analysis; funding acquisition; investigation; methodology; software; supervision; validation; visualization; writing – review and editing.

## FUNDING INFORMATION

SPC is supported by La Trobe University and the Max Planck Queensland Centre for the Materials Science of Extracellular Matrices. LC is supported by the European Union Horizon 2020 research and innovation program under the Marie Skłodowska‐Curie agreement (Grant No. 897218). JMC‐R is supported by an Australian Research Council DECRA Fellowship (DE240100501). This work was also funded by Australian Research Council Discovery grants (DP200103398, DP230101438, and DP240102532), Sea World Research and Rescue Foundation Inc., Ecological Society of Australia (The Holsworth Wildlife Research Endowment), an Australian Synchrotron Beamline Grant, Australia and Pacific Science Foundation, Save our Seas Foundation and La Trobe University, Australia.

## CONFLICT OF INTEREST STATEMENT

The authors declare no conflict of interest.

## PERMISSION TO REPRODUCE MATERIAL FROM OTHER SOURCES

Permission to reproduce previously published material has been granted and acknowledged.

## Data Availability

Data are available on request from the authors.
